# *Plasmodium* apicoplast tyrosyl-tRNA synthetase recognizes an unusual, simplified identity set in cognate tRNA^Tyr^

**DOI:** 10.1371/journal.pone.0209805

**Published:** 2018-12-28

**Authors:** Marta Cela, Caroline Paulus, Manuel A. S. Santos, Gabriela R. Moura, Magali Frugier, Joëlle Rudinger-Thirion

**Affiliations:** 1 UPR 9002 Architecture et Réactivité de l’ARN, Institut de Biologie Moléculaire et Cellulaire du CNRS, Strasbourg Cedex, France; 2 Department of Medical Sciences and Institute of Biomedicine - iBiMED, University of Aveiro, Aveiro, Portugal; Imperial College London, UNITED KINGDOM

## Abstract

The life cycle of *Plasmodium falciparum*, the agent responsible for malaria, depends on both cytosolic and apicoplast translation fidelity. Apicoplast aminoacyl-tRNA synthetases (aaRS) are bacterial-like enzymes devoted to organellar tRNA aminoacylation. They are all encoded by the nuclear genome and are translocated into the apicoplast only after cytosolic biosynthesis. Apicoplast aaRSs contain numerous idiosyncratic sequence insertions: An understanding of the roles of these insertions has remained elusive and they hinder efforts to heterologously overexpress these proteins. Moreover, the A/T rich content of the *Plasmodium* genome leads to A/U rich apicoplast tRNA substrates that display structural plasticity. Here, we focus on the *P*. *falciparum* apicoplast tyrosyl-tRNA synthetase (*Pf*-apiTyrRS) and its cognate tRNA^Tyr^ substrate (*Pf*-apitRNA^Tyr^). Cloning and expression strategies used to obtain an active and functional recombinant *Pf*-apiTyrRS are reported. Functional analyses established that only three weak identity elements in the apitRNA^Tyr^ promote specific recognition by the cognate *Pf*-apiTyrRS and that positive identity elements usually found in the tRNA^Tyr^ acceptor stem are excluded from this set. This finding brings to light an unusual behavior for a tRNA^Tyr^ aminoacylation system and suggests that *Pf*-apiTyrRS uses primarily negative recognition elements to direct tyrosylation specificity.

## Introduction

*Plasmodium falciparum* causes the most severe form of malaria in humans. Rapid constitutive growth and expansion of the parasite are highly dependent on the continuous synthesis of proteins in the cytosol and organellar compartments [1]. Indeed, *Plasmodium* contains three genomes: nuclear, apicoplast (a relic chloroplast) and mitochondrial. These genomes require dedicated translation machineries to function, even if translation has not yet been explicitly demonstrated in the mitochondria. The *Plasmodium* apicoplast is essential and is involved in the synthesis of fatty acids, isoprenoid precursors and heme [2]. It has a 35 kb circular genome encoding 30 protein genes, all of which are involved in apicoplast transcription and translation including, for example, the subunits of RNA polymerase, elongation factor Tu, several ribosomal proteins, as well as ribosomal RNAs and transfer RNAs (tRNAs) [3]. However, most proteins essential for apicoplast functions are encoded by the nuclear genome and are imported into the organelle after translation. The *P*. *falciparum* genome contains more than 450 proteins genes with targeting signals for the apicoplast [4], among them are the apicoplast aminoacyl-tRNA synthetases (aaRSs), dedicated to the specific aminoacylation of apicoplast tRNAs with their corresponding amino acids.

Generally, there are 20 different aaRSs in each protein translation compartment, with each enzyme responsible for aminoacylating a specific tRNA isoacceptor set with the matching, cognate amino acid [5]. In *Plasmodium*, 37 nuclear genes encode 36 aaRSs (the cytosolic phenylalanyl-tRNA synthetase being a heterotetramer) [1,6]: 17 are exclusively cytosolic, 20 possess an apicoplast targeting sequence (4 of these, alanyl-, glycyl-, threonyl- and cysteinyl-tRNA synthetases, are targeted to both the apicoplast and the cytosol [7,8]) and only one is putatively mitochondrial [9].

Although all aaRSs perform the same reaction, namely the attachment of an amino acid to the 3’ end of their cognate tRNA(s), they are structurally different. Depending on the architecture of the active site and the tRNA binding mode, aaRSs are divided into two structural classes, with 10 enzymes in each class [5,10,11]. In addition to the catalytic domain, they typically contain a tRNA-binding domain, which often recognizes the anticodon triplet of the tRNA. This organization generally holds true for organellar aaRSs, with some variations in size, oligomerization, and function [12]. Apicoplast aaRSs are evolutionarily conserved enzymes, yet previous studies have highlighted the presence of numerous uncharacterized insertions, which therefore increase their sizes significantly [6]. The presence of these insertions could explain why apicoplast aaRSs are poorly studied *in vitro*, despite their potential as therapeutic targets for the development of new anti-malarial drugs [13–15].

It is generally accepted that the fidelity of protein synthesis depends mostly on the formation of correct aminoacylation of tRNAs, the reaction catalyzed by the aaRSs [5,16]. Thus, a given aaRS selects one amino acid from the 20 canonical amino acids and one isoacceptor set of tRNAs from all tRNAs. The specificity of this process is mainly governed by identity elements scattered throughout tRNA structures. Positive identity elements are identified as nucleosides or structural motifs that are recognized in a specific manner by the cognate aaRS to target that tRNA for aminoacylation. Negative identity elements play an essential role in preventing the binding of non-cognate aaRSs to a given tRNA. tRNA identity sets have been characterized for most bacterial aminoacylation systems, but fewer examples have been studied in depth in archaeal, eukaryal or organellar systems [17,18]. Moreover, in view of the conserved, canonical cloverleaf structure of tRNAs, the nature of these identity elements are generally preserved across evolution with only a few exceptions. In particular, the tyrosine aminoacylation system is characterized by a robust phylogenetic barrier that limits cross-reactivity between partners from different species. For example, *E*. *coli* TyrRS does not aminoacylate eukaryotic tRNA^Tyr^ and *vice-versa* [19].

Guided by the above background, we set out to characterize the *P*. *falciparum* apicoplast TyrRS/tRNA^Tyr^. We characterized the structural features required to specifically aminoacylate the apicoplast tRNA^Tyr^ (*Pf*-apitRNA^Tyr^, encoded in the apicoplast genome) using the apicoplast tyrosyl-tRNA synthetase (*Pf*-apiTyrRS, encoded in the nuclear genome). These efforts required: (i) the engineering of the *Pf*-apiTyrRS gene to produce a soluble recombinant protein containing two *Plasmodium*-specific insertions; and (ii) confirmation that the A/U rich *Pf*-apitRNA^Tyr^ transcript could be properly folded. Then, a systematic study of nucleotide replacements in tRNA, including a comparative examination of their activities, established the rules that govern recognition of *Pf*-apitRNA^Tyr^ by its cognate TyrRS. Our analysis demonstrates that the identity elements in *Pf*-apitRNA^Tyr^ are unusually reduced in strength and number. These results reveal that the identity elements of the apicoplast tyrosine aminoacylation system are both distinct and minimalistic in comparison to those that have been conserved evolutionarily elsewhere.

## Material and methods

### Cloning and purification of *P*. *falciparum* apicoplast TyrRS

The genomic sequence of *Pf*-apiTyrRS was retrieved from PlasmoDB [20] by sequence homology with the human mitochondrial TyrRS (EAW88518.1, *Hs*-mitoTyrRS) and *Thermus thermophilus* TyrRS (AEG33811.1) [6]. The gene (PF3D7_1117500) codes for a 561 amino acid protein. The *Pf*-apiTyrRS gene was amplified by PCR from a *P*. *falciparum* cDNA library (provided by Dr. H. Vial, Montpellier), sequenced, and cloned into pQE30 (Qiagen) to produce a protein with a 6-histidine fusion tag at its N-terminus. This plasmid expresses the *Pf*-apiTyrRS recombinant protein without its N-terminal apicoplast targeting signal and covers amino acids 25 to 561 (S1 Fig).

In addition to cloning of the endogenous *P*. *falciparum* nucleotide sequence of *Pf*-apiTyrRS (Endo), two additional nucleotide sequences, both encoding the same amino acid chain, were designed (S2 Fig). In the first, we produced an optimized version (Opt) of the *Pf*-apiTyrRS gene based on *Escherichia coli* codon usage (designed by Genscript) and a harmonized version (Harm) of the gene using the ANACONDA software [21,22]. Both genes were synthesized by GenScript (https://www.genscript.com) and cloned into the pQE30 plasmid. Furthermore, a truncated variant of the Harm *Pf*-apiTyrRS gene, lacking the C-terminal S4-like domain (residues 461 to 561) was cloned into pQE70 with a C-terminal 6-His tag.

Overexpression of all recombinant proteins was performed at 18 °C overnight in LB medium containing 0.1 mg/mL ampicillin and 1 mM IPTG (Isopropyl β-D-1-thiogalactopyranoside), and the purification of 6 His-tagged proteins was accomplished on Ni-NTA resin according to the manufacturer’s instructions (Qiagen). Purified enzymes were dialyzed against 25 mM HEPES-KOH pH 7.5, 25 mM KCl, 50% glycerol and were kept at -20 °C until use. Proteins were quantified through absorbance measurements and their enzymatic activities were assessed by *in vitro* aminoacylation of native *E*. *coli* tRNA^Tyr^. Gel filtration analysis was performed on a Superdex 200 increase 10/3000 GL column (GE Healthcare) in 50 mM potassium phosphate buffer pH 7.5, 150 mM KCl, 10% glycerol and 1 mM EDTA.

### Sequence analysis

Sequence alignments were computed with Tcoffee [23], and CLUSTALW [24] softwares. The prediction of *Pf*-apiTyrRS secondary structure was achieved with the PredictProtein software [25]. The PlasmoAP algorithm [20] confirmed the presence of an apicoplast targeting signal and predicted the cleavage site. The *E*. *coli* codon usage database was from [26].

### Preparation of tRNA molecules

Native *E*. *coli* tRNA^Tyr^ (Genbank CP010229.1) was purchased from Sigma-Aldrich. Transcripts corresponding to wild-type (PF3D7_API00700) and mutated *Pf*-apitRNA^Tyr^ were synthesized by *in vitro* transcription using the transzyme method [27]. tRNA transcripts were obtained by *in vitro* transcription of synthetic genes cloned in pUC 119 and linearized with *Bst*NI (to produce a 3’ CCA sequence after transcription). Each of these genes corresponds to the T7 RNA polymerase promoter, followed by a hammerhead ribozyme, and the tRNA gene sequences. *In vitro* transcriptions were performed in reaction mixtures containing 40 mM Tris-HCl pH 8.1, 22 mM MgCl_2_, 5 mM dithioerythrytol, 0.1 mM spermidine, 4 mM each nucleoside triphosphate, 5 mM GMP, 50 ng/mL linearized plasmid and 5 ng/mL T7 RNA polymerase. Transcription mixtures were incubated for 3 h at 37°C and reactions were stopped by phenol extraction. Auto-catalytically cleaved transcripts correctly ending with the CCA-sequence were purified by preparative electrophoresis on 12% polyacrylamide denaturing gels (8 M urea) followed by electroelution (Schleicher & Schuell apparatus). Concentration of tRNA transcripts was determined by absorbance at 260 nm. We similarly produced transcripts corresponding to the wild-type *P*. *falciparum* apicoplast tRNA^Ser^_GCU_ (PF3D7_API00800, *Pf*-apitRNA^Ser^).

### Procedures for structural analysis of free and TyrRS-complexed tRNAs

Lead and enzymatic probing were performed as in [28] with the following details described here:

Lead probing: 1 μM of 5’-labeled *Pf*-apitRNA^Tyr^ wild-type transcript (80,000 cpm) was incubated in 50 mM Tris-acetate pH 7.5, 5 mM magnesium acetate, 50 mM potassium acetate. A solution of Pb(OAc)_2_, freshly prepared in H_2_O, was added to reach final concentrations of 1, 3, 6 and 10 mM. The samples were incubated for 6 min at 25 °C.

Enzymatic probing: 1 μM of 5’-labeled *Pf*-apitRNA^Tyr^ transcript (80,000 cpm) was incubated in 20 mM MgCl_2_, 100 mM NaCl, 50 mM Hepes-NaOH pH 7.5 in the presence of T1 (0.2 U), S1 (5.4 U) and V1 (7x10^-4^ U) nucleases for 10 min at 25 °C. For S1 probing, 1 mM ZnCl_2_ was added. A control experiment without nuclease was performed in parallel.

All reactions were stopped by the addition of 20 μl of Stop Mix (0.6 M NaOAc, 4 mM EDTA, 0.1 mg/mL total tRNA, and 1 μg glycogen) and ethanol precipitated. The pellets were washed twice with 70% ethanol, vacuum-dried, dissolved in gel loading mix (90% formamide, 0.5% EDTA, 0.1% xylene cyanol and 0.1% bromophenol blue), heated 2 min at 90 °C, and then loaded on a 12% denaturing gel. In parallel, T1 nuclease and alkaline hydrolysis reactions were performed under denaturing conditions to accurately assign the bands in each gel.

Footprinting assays (10 μL) were performed under the same conditions as above in the absence or presence of 5.7 μM *Pf*-apiTyrRS. The tRNA/TyrRS complex was incubated for 6 min at 25 °C, before 0.2 U T1 or 5.4 U S1 (supplemented with 1 mM ZnCl_2_ for S1 cleavage) were added. Incubation was continued for 8 min at 25 °C and the reactions were stopped by phenol extraction. After precipitation, the pellets were treated as described above.

### Aminoacylation assays

Tyrosylation assays were performed at 37 °C in 50 mM Hepes-KOH (pH 7.6), 25 mM KCl, 12 mM MgCl_2_, 2.5 mM ATP, 0.2 mg/ml bovine serum albumin, 1 mM spermine, 10 μM L-[^14^C]-tyrosine at 486 mCi/mmol (Perkin Elmer), and appropriate amounts of either native or transcribed tRNA^Tyr^ (0.5 to 10 μM). *Pf*-apiTyrRS (50 to 100 nM, diluted in 100 mM Hepes-NaOH pH 7.4, 1 mM DTT, 5 mg/mL BSA, and 10% glycerol) was added to start the reaction [29]. The apparent kinetic parameters were determined from Lineweaver-Burk plots.

## Results

### Identification of the *P*. *falciparum* apicoplast TyrRS gene

**Fig 1:**
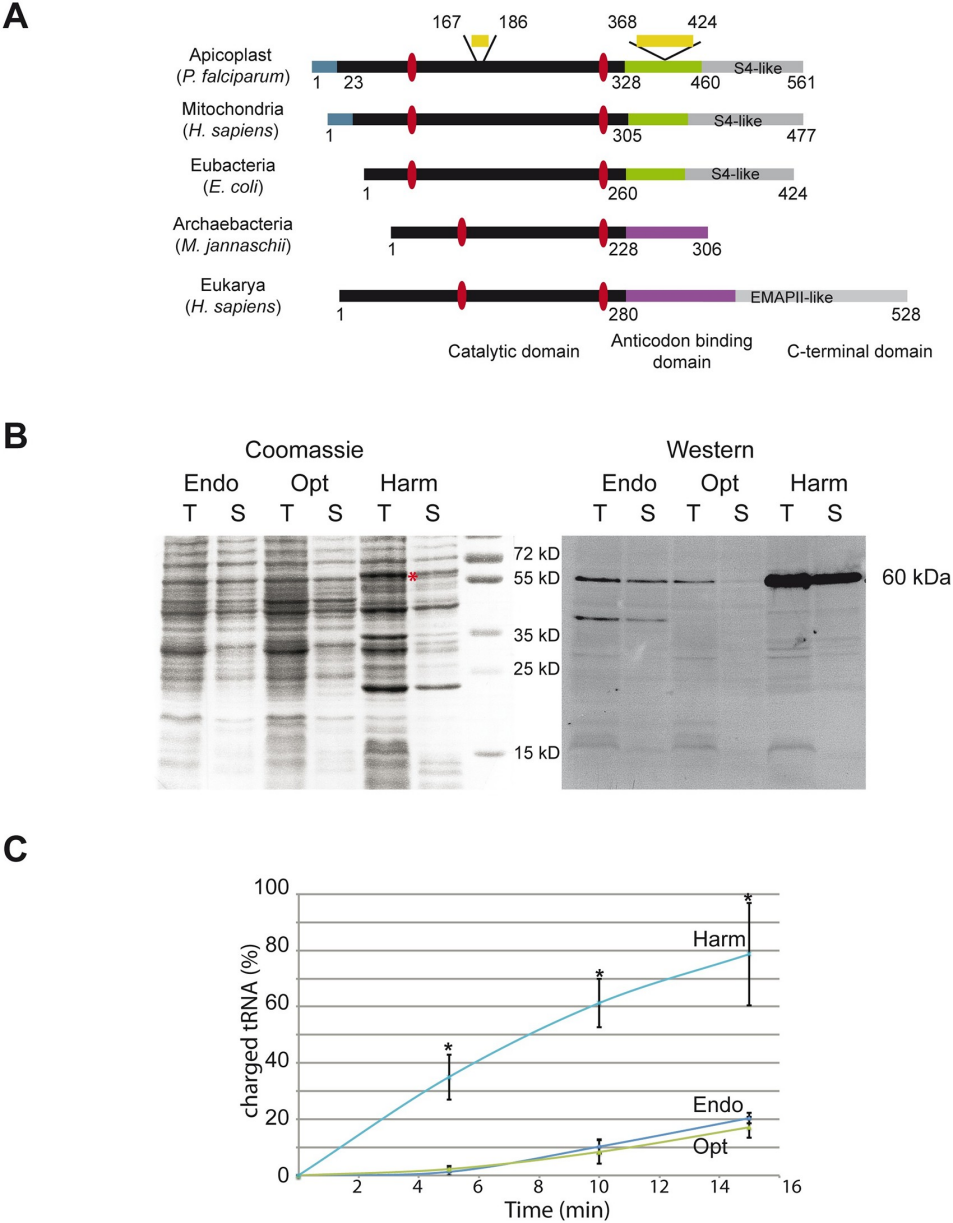
Expression of a soluble and active *Pf*-apiTyrRS in *E*. *coli*. (A) The overall organization of *Pf*-apiTyrRS, compared to TyrRSs from humans (mitochondria and cytosol), bacteria, and archaea. Each structural domain is given in a specific color: blue for mitochondrial and apicoplast targeting signals, black for catalytic domains, green for the canonical α-helical anticodon-binding domains, purple for anticodon-binding domains homologous to TrpRS [31], and grey for additional C-terminal domains (S4-like in bacteria and organelles; EMAPII-like in vertebrates [32]). *Plasmodium*-specific idiosyncratic insertions are shown in yellow. Red dots indicate the position of the signature sequences present in the catalytic domains of class I aaRSs. (B) Coomassie-stained gel (loading control) and the corresponding Western blot show *Pf*-apiTyrRS expressed from the *Plasmodium* wild-type nucleotide sequence (Endo), the optimized gene sequence for *E*. *coli* expression (Opt), and the harmonized gene sequence (Harm). T stands for Total extract, and S stands for Soluble extract (supernatant of the centrifuged total extract). On the coomassie-stained gel, overexpression of the Harm *Pf*-TyrRS is indicated with a red asterisk. Additional bands potentially correspond to degradation products. On the Western Blot, 6-His tagged proteins were specifically detected with a mouse anti-penta-his tag antibody (Qiagen). (C) Aminoacylations of *E*. *coli* tRNA^Tyr^ (0.8 μM) performed with *Pf*-apiTyrRSs (100 nM) produced from the three gene variants were measured after 5, 10 and 15 min incubation. Controls (without tRNA) were substracted. Results are an average of three independent experiments. *significant at p<0.05 (t-test) (S1 Table).

We identified the gene coding for the 561 amino acid *Plasmodium Pf*-apiTyrRS (PF3D7_1117500) containing an N-terminal catalytic domain, an anticodon binding domain at its C-terminus, and a putative signal sequence for apicoplast targeting [6] (Fig 1A). The Rossman-fold-containing catalytic domain (amino acids 1–328) presents both class I aaRS specific motifs (HNGL and KYSKS). As expected, the C-terminal domain of *Pf*-apiTyrRS presents the typical α-helical domain (amino acids 329–460) and the S4-like region (amino acids 461–561) that are both specific features found only in bacterial and mitochondrial TyrRSs (reviewed in [30]).

The PlasmoAP algorithm [20] that predicts apicoplast targeting signals indicated that the N-terminal extremity of *Pf*-apiTyrRS "very likely" contains an apicoplast targeting signal and secondary structure predictions positioned this targeting sequence within an α-helix (S1 Fig). These information led us to the deletion of the first 24 amino acids from the N-terminus in the recombinant *Pf*-apiTyrRS.

*Pf*-apiTyrRS is longer than its prokaryotic homologs because it contains two insertions of 19 and 56 amino acids in the catalytic and the anticodon-binding domains, respectively (Fig 1A). Sequence alignments with seven other *Plasmodium* apicoplast TyrRSs (*P*. *reichenowi*, *P*. *vivax*, *P*. *knowlesi*, *P*. *gallinaceum*, *P*. *yoelii*, *P*. *chabaudi*, *P*. *berghei*) revealed conserved locations for these insertions, while their sizes and sequences vary significantly (S1 Fig). The insertion located in the anticodon-binding domain is characterized by single amino acid repeats [33]. Indeed, this insertion is composed of 30% asparagine residues.

### Production of a functional recombinant *P*. *falciparum* apicoplast TyrRS

*Pf*-apiTyrRS_25-561_ could be expressed in *E*. *coli* directly from the *P*. *falciparum* wild-type nucleotide sequence (Endo), but the affinity-purified yield of soluble protein was poor (<1.5 mg protein per liter culture). Alternative strategies were used to improve the production of soluble *Pf*-apiTyrRS_25-561_. Two different synthetic gene sequences both encoding the same wild-type *Pf*-apiTyrRS_25-561_ were cloned (S2 Fig). In one case (Opt), the coding DNA sequence was changed using standard codon optimization rules for expression in *E*. *coli* (GenScript). In the second case, the DNA sequence was “harmonized” (Harm) using the bioinformatics application for gene primary structure analysis ANACONDA. This program uses statistical methods to analyze not only the codon usage but also the codon context (degree of association, context, and clustering) on a genomic scale [21,22]. In other words, it takes into account the rules governing the evolution of codon bias in *P*. *falciparum* to design a new nucleotide sequence adapted to the *E*. *coli* translational apparatus. The main differences between Opt and Harm were at the level of leucine codons, which were all substituted with CTA in the harmonized gene (S2 Fig); CTA is the rarest amongst the six leucine codons (0.385%) in *E*. *coli* [26].

Gene expression and protein purification using the Opt gene did not change the expression of the protein significantly compared to the endogenous *Plasmodium* DNA sequence (Endo), however, the solubility of *Pf*-apiTyrRS_25-561_ was reduced (Fig 1B) and purification yields were low (<0.4 mg protein per liter culture). The best expression yields were obtained with the Harm gene, which yielded nearly 3-fold more *Pf*-apiTyrRS after affinity purification (about 4 mg protein per liter culture) (S3A and S3B Fig). Furthermore, comparative aminoacylation assays (Fig 1C) using native *E*. *coli* tRNA^Tyr^ demonstrated that the enzymes produced from these three plasmids were not functionally equivalent. Indeed, the Harm *Pf*-apiTyrRS is significantly more efficient in aminoacylation than the other two preparations. This observation indicates that gene harmonization not only increased the solubility and hence the purification yields of *Pf*-apiTyrRS_25-561_, but also improved the correct folding of the recombinant protein. Indeed, the purified Harm *Pf*-apiTyrRS_25-561_ protein elutes as a major peak of about 160 kDa on a gel filtration column, suggesting that it forms the expected 118 kDa homodimer (S3C Fig). Thus, *Pf*-apiTyrRS_25-561_ expressed from the harmonized construct was used to determine the kinetic parameters of all *Pf*-apitRNA^Tyr^ mutants.

*Pf*-apiTyrRS displays an S4-like domain at its C-terminus, specific to bacterial and mitochondrial TyrRSs (Fig 1A). In general, the elimination of this domain increases solubility, while decrease the enzyme’s affinity for tRNA^Tyr^ (*e*.*g*. [34]), since recognition of the tRNA variable region is disrupted [35]. In contrast to these prokaryotic-type TyrRSs, the truncation of the S4-like domain of *Pf*-apiTyrRS led to an inactive enzyme that does not catalyze the first step of the aminoacylation reaction (tyrosine activation in the presence of ATP as measured by ATP/PPi exchange assays (S3D Fig)). This result suggests that, unlike all other known TyrRSs, deletion of the C-terminal domain of *Pf*-apiTyrRS destabilizes the folding of the N-terminal catalytic site.

### Sequence peculiarities in *P*. *falciparum* apicoplast tRNA^Tyr^

**Fig 2:**
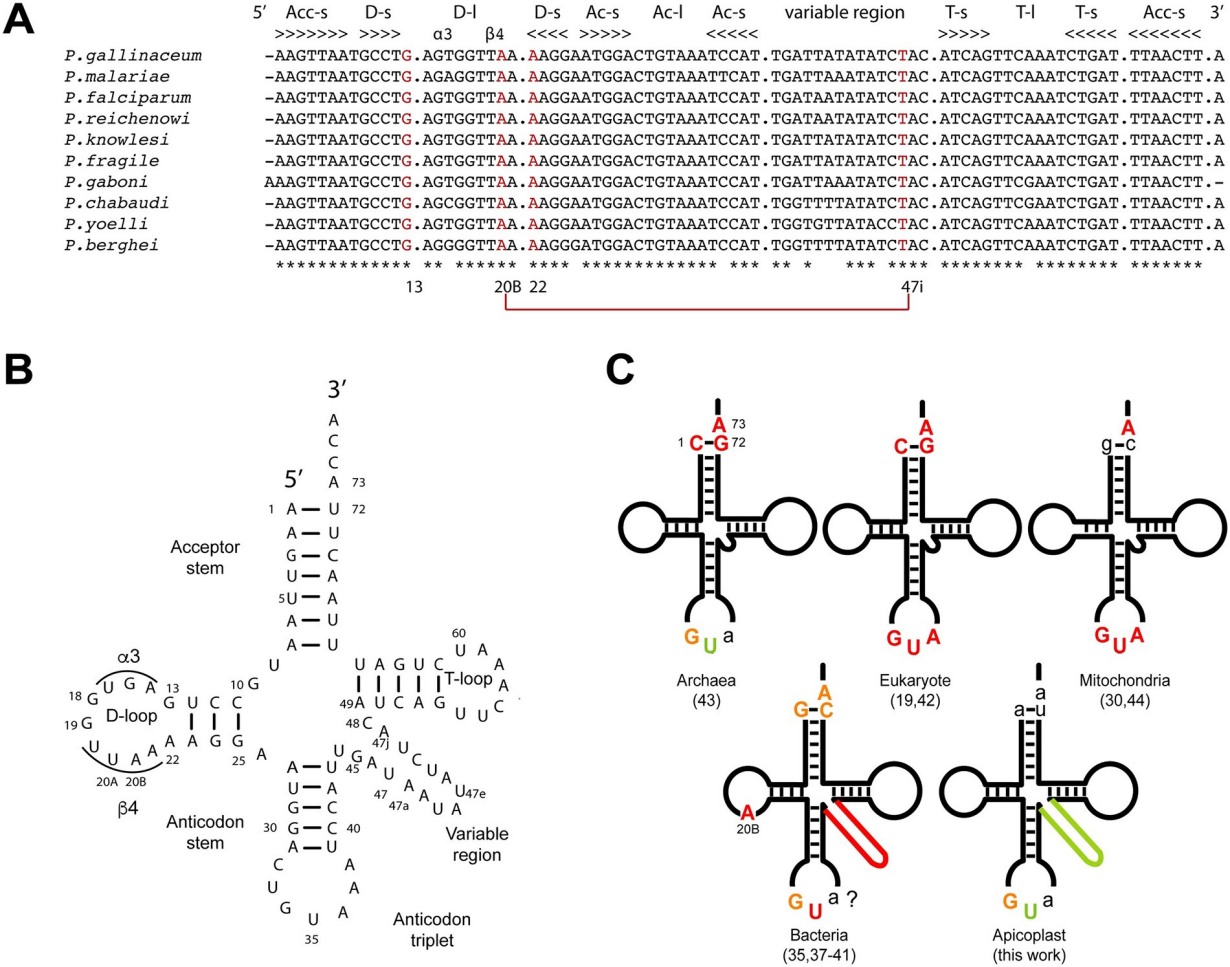
Sequence of *Pf*-apitRNA^Tyr^ and evolution of tyrosine identity. (A) Comparison of tyrosine-specific apicoplast tRNAs belonging to different *Plasmodia* species. The domains of the cloverleaf structure are indicated at the top: Acc-s refers to the acceptor-stem; D-s refers to the D-stem; D-l refers to the D-loop; Ac-l refers to the anticodon-loop; T-s refers to the T-stem; and T-l refers to the T-loop. Structural elements or nucleotides involved in specific tertiary interactions are highlighted in red. (B) Secondary structure of *Pf*-apitRNA^Tyr^. The architectural organization of the D-loop (α and β domains on both sides of residues G_18_G_19_) is indicated. Notice the non-canonical G_13_-A_22_ base pair located at the end of the D-arm. The tRNA is numbered according to [36]. (C) 2D schematic structures of tRNA^Tyr^ showing the residues involved in tyrosylation in different phylae [19,30,37–44]. The residues involved in tyrosine identity are explicitly given in uppercase. The strengths of the tyrosine identity elements are indicated by colors: red (loss in catalytic efficiency >100-fold compared to the wild-type transcript), orange (loss between 10- and 100-fold) and green (loss between 5- and 10-fold). Lowercase letters are given to highlight conservation of the residues in the anticodon triplet and at positions 1–72 and 73 in the acceptor stem, despite their exclusion from the identity set. The question mark shows that the importance of position 36 has not been tested in bacteria.

The natural substrate for *Pf*-apiTyrRS is the only tRNA^Tyr^ encoded by the apicoplast genome (Fig 2A and 2B). *Pf*-apitRNA^Tyr^ is composed of 68% A/U residues (both in stems and loops) reflecting the rich A/T composition of the *P*. *falciparum* genome [3]. *Pf*-apitRNA^Tyr^ displays (i) the phylogenetically conserved A_73_ discriminator base; (ii) an α3-β4 D-loop with a non-canonical G_13_-A_22_ base pair in the D-stem; and (iii) is a class 2 tRNA (like bacterial tRNA^Tyr^s), defined as tRNAs with a large variable region (Fig 2B). Interestingly, *Pf*-tRNA^Tyr^ is characterized by the presence of an A_1_-U_72_ base pair at the top of the acceptor stem. This base pair is conserved in *Plasmodium* apicoplast tRNA^Tyr^ sequences referenced in EupathDB [20] (some of which are displayed in Fig 2A), while tRNA^Tyr^ from bacteria, mitochondria, and chloroplasts are characterized by a G_1_-C_72_ base pair and tRNA^Tyr^ from archaea and eukarya contain a C_1_-G_72_ base pair (Fig 2C).

### Cloverleaf folding of P. falciparum apicoplast tRNA^Tyr^

**Fig 3:**
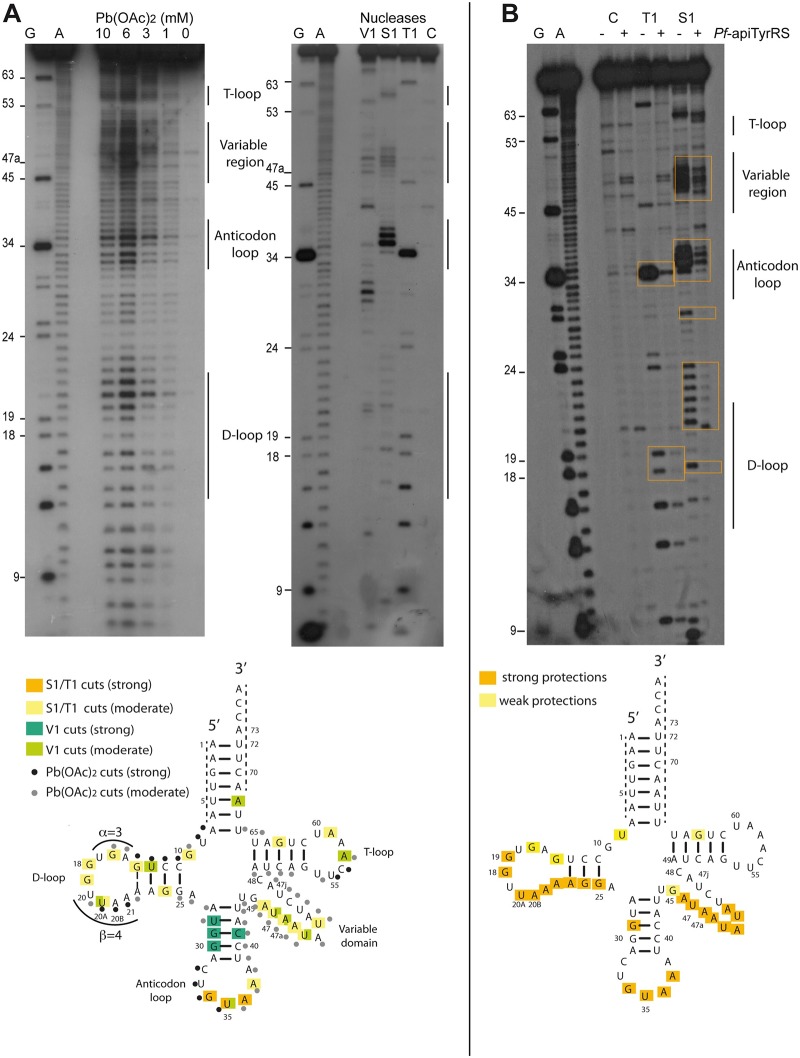
Probing of *Pf*-apitRNA^Tyr^ transcript. (A) Autoradiograms from probing experiments analyzing 5’-labeled *Pf*-apitRNA^Tyr^ transcript with Pb(OAc)_2_ (left panel), RNases V1, S1, and T1 (right panel). The final concentrations of Pb(OAc)_2_ are indicated at the top of the autoradiogram. The G lane corresponds to a denaturing RNase T1 ladder and lane A to an alkaline ladder. The C lane shows the control experiment performed with untreated transcript. The locations of D-, anticodon- and T- loops, as well as the large variable domain, are noted. Results are summarized on the *Pf*-apitRNA^Tyr^ cloverleaf structure. The tRNA is numbered according to [36]. Nucleotides at the 3’- and 5’- ends, which cannot be analyzed are indicated by dotted lines on the tRNA structure. (B) Autoradiogram corresponding to footprinting experiments: the 5’-labeled *Pf*-apitRNA^Tyr^ transcript was incubated with S1 and T1 nucleases in the absence (-) or presence (+) of *Pf*-apiTyrRS. The strongest RNase protections which confirm the sites of interaction between the tRNA^Tyr^ transcript and *Pf*-apiTyrRS are framed in orange. Controls (C) were performed without nucleases. G and A indicate T1 and alkaline ladders, respectively. *Pf*-apitRNA^Tyr^ residues that are protected from nucleases in the presence of *Pf*-apiTyrRS are indicated in orange (strong protection) and yellow (weak protection) on the *Pf*-apitRNA^Tyr^ cloverleaf structure.

The 87 nucleotide *Pf*-apitRNA^Tyr^ was produced as a transcript lacking modified bases. Probing experiments were performed with Pb(OAc)_2_ and nucleases (Fig 3A) to verify that the high proportion of A and U residues together with the absence of posttranscriptional modifications do not hinder the formation of the canonical cloverleaf fold. S1 and T1 nucleases and Pb(OAc)_2_ are specific for single-stranded regions, whereas the V1 nuclease recognizes double-stranded and highly structured regions. In Fig 3A, the similarities between RNAse T1 profiles in native (T1) and denaturing (G) conditions suggest that the *Pf*-tRNA^Tyr^ transcript is flexible. This hypothesis was confirmed by the pattern of lead cleavage positions, which occur throughout the sequence, with the strongest cuts concentrated in the loops and the variable region.

As expected for a cloverleaf fold, the strongest S1 and V1 cuts are only found in the anticodon-loop and the anticodon-stem, respectively, indicating that this portion of the transcript is indeed folded. Moreover, the moderate accessibilities of A_57_ and U_20A_ to V1 cleavage confirm the presence of tertiary interactions between the T- and D- loops, whereas conflicting V1 and S1 cuts in the variable region suggest a fluctuating structure in this domain. Altogether, these probing data are in agreement with a cloverleaf fold and indicate intrinsic structural plasticity, reminiscent of what has been observed for some mitochondrial tRNAs [28,45]. Moreover, the correct folding of *Pf*-apitRNA^Tyr^ was also confirmed by aminoacylation assays with the homologous Harm *Pf*-apiTyrRS_25-561_, since tyrosylation of *Pf*-apitRNA^Tyr^ occurred with the same catalytic efficiency as with the native *E*. *coli* tRNA^Tyr^ (Table 1).

**Table 1:** Tyrosylation kinetic parameters of *E*. *coli* tRNA^Tyr^ and wild-type or mutated *Pf*-apitRNAs by *Pf*-apiTyrRS.

	K_m_ (μM)	S.D. *K*_*m*_	*k*_*cat*_ (10^-3^sec^-1^)	S.D. *k*_*cat*_	*k*_*cat*_*/K*_*m*_ (10^-3^sec^-1^.μM^-1^)	p-value	L^a^ (x-fold)
*E*. *coli* tRNA^Tyr^	0.9	0.2	12.0	5.1	14.1	0.197	1.5
*Pf*-apitRNA^Tyr^	0.8	0.3	16.0	4.0	20.6		1.0
*Pf*-apitRNA^Ser^	nd		nd		-	-	>1000
**Acceptor stem**							
G_73_	1.0	0.3	9.8	5.4	9.2	0.021	2.2*
C_73_	0.5	0.2	4.1	0.5	8.9	0.147	2.3
U_73_	0.8	0.3	4.0	1.7	4.9	0.019	4.2
G_1_-C_72_	1.2	1.0	10.3	3.0	17.4	0.793	1.2
C_1_-G_72_	0.3	0.7	4.5	2.3	10.8	0.007	1.9*
C_2_-G_71_/U_3_-A_70_	0.4	0.2	3.0	0.6	8.0	0.019	2.6*
**Anticodon triplet**							
C_34_	6.2	3.4	3.2	1.6	0.6	0.014	36.0*
G_35_	0.7	0.2	13.3	6.5	19.8	0.786	1.0
C_35_	1.6	0.5	4.2	1.6	2.7	0.012	7.7*
A_35_	1.7	0.7	4.5	0.8	3.0	0.008	6.8*
C_36_	0.6	0.3	3.5	0.8	6.8	0.015	3.0*
U_36_	1.0	0.3	6.7	1.5	7.0	0.021	3.0*
G_36_	1.6	0.4	9.7	2.2	7.0	0.026	3.0*
Ser (C_35_U_36_)	1.3	0.3	1.3	0.4	1.1	0.014	19.1*
**Variable region (Vr)**							
ΔVr	1.8	1.0	4.5	0.9	2.9	0.014	7.1*
SerVr	0.8	0.4	5.6	2.3	7.6	0.013	2.7*
**D-loop (D-l)**							
U_20B_	0.9	0.4	8.2	3.0	12.6	0.353	1.6
SerVr+D-l	1.0	0.3	7.4	2.4	8.1	0.047	2.1*

*K*_*m*_ and *k*_*cat*_ values correspond to means from three independent experiments or more (Table S1) with the indicated error (S.D.). Mean values of *k*_*cat*_/*K*_*m*_ and p-values were calculated independently.

*significant at p<0.05 (t-test).

^a^L values correspond to losses of catalytic efficiency relative to wild-type *Pf*-apitRNA^Tyr^. nd indicates not detectable.

### Extensive recognition of *P*. *falciparum* apicoplast tRNA^Tyr^ by its cognate TyrRS

S1 and V1 nucleases were used in footprinting experiments (Fig 3B) to detect the protected regions of *Pf*-apitRNA^Tyr^ in the presence of *Pf*-apiTyrRS_25-561_. The anticodon-loop and the variable domain of the *Pf*-apitRNA^Tyr^ transcript are both strongly protected from RNase cleavage and are consistent with what has been observed in the crystallographic structure of the *T*. *thermophilus* TyrRS/tRNA^Tyr^ complex [35]. However, protection patterns detected in the *Pf*-apitRNA^Tyr^ D-domain are dissimilar to the bacterial recognition pattern [35]. The addition of *Pf*-apiTyrRS_25-561_ protected the *Pf*-apitRNA^Tyr^ D-loop from nuclease cleavage, as a consequence either of a direct contact with the synthetase, or of an indirect effect due to a conformational change in the tRNA bound to the enzyme.

### Looking for identity elements in *P*. *falciparum* apicoplast tRNA^Tyr^

TyrRS displays species-specific tRNA recognition (summarized in Fig 2C with the references therein). The tRNA^Tyr^ A_73_ discriminator base and the G_34_ anticodon nucleotide are universally important for TyrRS recognition. Anticodon nucleotides U_35_ and A_36_ also contribute to tyrosylation identity, yet with varying strengths in eukarya, bacteria, and mitochondria, and are marginal in archaea. Notably, the G_1_-C_72_ identity base-pair, located at the top of the acceptor stem in bacterial tRNA^Tyr^, is replaced by a C_1_-G_72_ identity base pair in eukarya tRNA^Tyr^. Finally, the large variable region is unique to bacterial tRNA^Tyr^ and is essential for tyrosylation.

**Fig 4:**
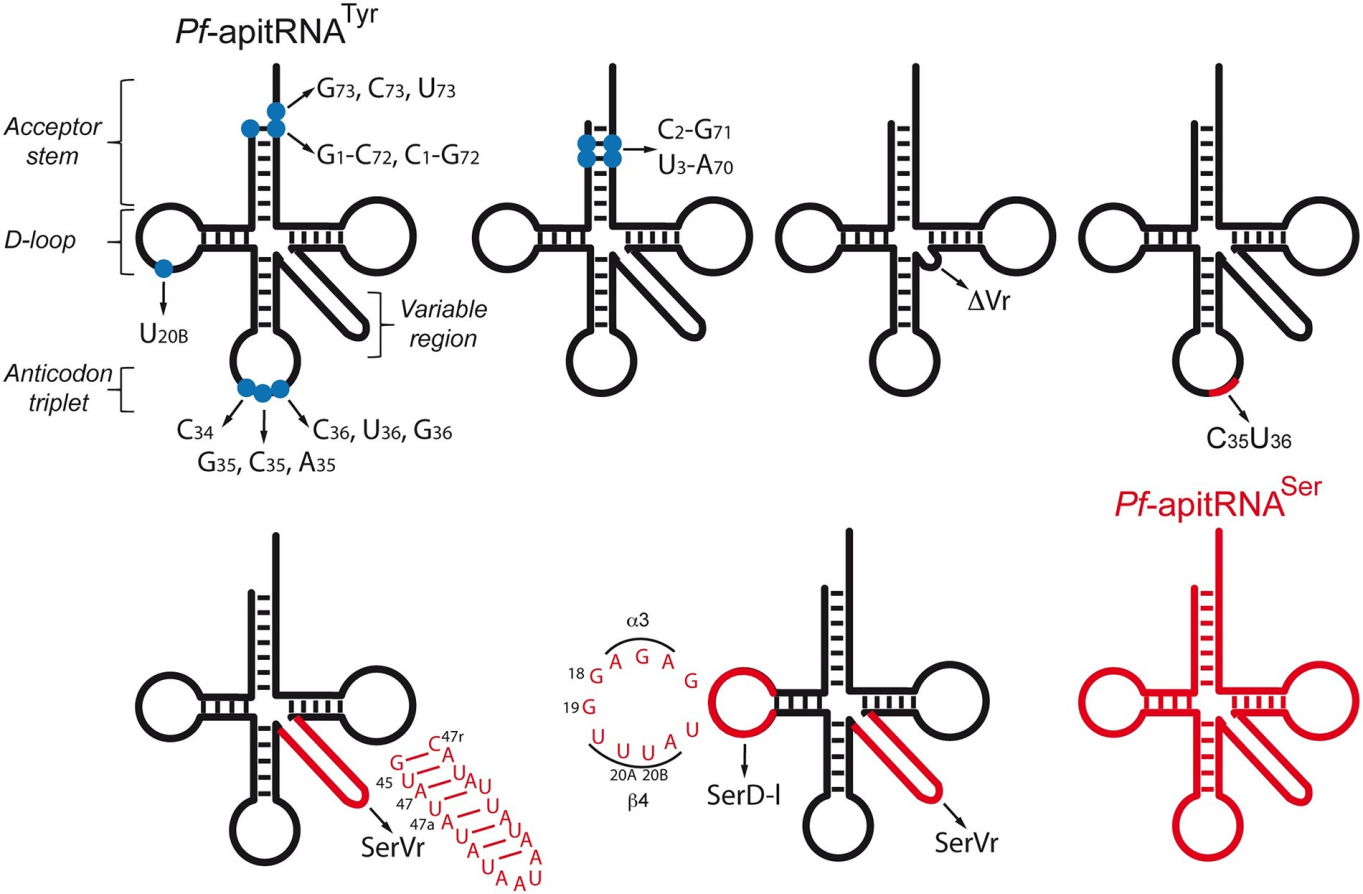
2D schematic structures of *Pf*-apitRNA^Tyr^ summarizing the locations and the nature of the mutated residues. Mutated sequences are indicated by blue dots and mutations are listed. In addition to the single mutations shown, 10 residues were deleted from the variable region (ΔVr: from 47 to 47i). The 2D schematic structure of *Pf*-apitRNA^Ser^ and the serine-specific features introduced into *Pf*-apitRNA^Tyr^, such as dinucleotide C_35_U_36_, the variable region (SerVr), and the D-loop plus the variable region (SerVr+D-l) are shown in red.

We chose to elucidate the identity determinant set for *Pf*-apitRNA^Tyr^ with *Pf*-api-TyrRS_25-561_. Eighteen mutants were designed to test the extremity of the acceptor stem (discriminator base 73 and the 1–72 base-pair), the anticodon triplet, and the variable region for their importance for tyrosylation (Fig 4). The kinetic parameters for tyrosylation were determined and compared to those obtained for the wild-type *Pf*-apitRNA^Tyr^ transcript (Table 1).

#### Acceptor stem

Unexpectedly, replacement of the A_73_ discriminator base by G, C or U reduced tyrosylation only 2.2 to 4.2-fold, suggesting that these mutations do not affect the recognition of tRNA^Tyr^ by *Pf*-apiTyrRS, but slightly modulate the structure of the tRNA near the catalytic site. Indeed, it has been shown that a pyrimidine residue at position 73 might influence the stability of the acceptor stem extremity [46]. Unlike the vast majority of tRNA^Tyr^ isoacceptors, where the first base pair in the acceptor stem is G_1_-C_72_ (in bacteria) or C_1_-G_72_ (in archaea and eukarya), all *Plasmodia* apicoplast tRNA^Tyr^ contain a conserved A_1_-U_72_ (Fig 2A). A_1_-U_72_ was therefore changed to G-C and C-G, (Fig 4 and Table 1). *Pf*-apiTyrRS_25-561_ aminoacylates the wild-type transcript, the G_1_-C_72_ and the C_1_-G_72_ variants with similar kinetic values, indicating that the first base pair of the acceptor stem is not part of this system’s tyrosine identity set, providing the second example (with the human mitochondrial TyrRS) of a TyrRS lacking specificity to base pair 1–72 [44]. We hypothesized that a different base pair in the acceptor stem might have replaced 1–72 in the identity set, so we mutated positions 2–71 and 3–70 (Fig 4 and Table 1). The recombinant *Pf*-apiTyrRS aminoacylated these variants with kinetic parameters similar to those of the wild-type transcript, characterized by a loss in efficiency of only 2.6-fold. Together, these data show the absence of tyrosine identity elements in the acceptor stem and suggest that *Pf*-apiTyrRS only recognizes the ribose-phosphate backbone in this region of the tRNA molecule.

#### Anticodon triplet

Residues 34, 35 and 36 were individually mutated in *Pf*-apitRNA^Tyr^. Only the variant where C_34_ replaced G_34_ showed a substantial loss in tyrosylation (36-fold). Mutations at position 35 affected catalytic efficiency by 1 to 7.7-fold and mutations at position 36 had virtually no effect on activity (3.0-fold) (Table 1). Moreover, converting the tyrosine anticodon to a serine GCU anticodon (mutant Ser C_35_U_36_) only leads to a moderate but significant decrease of 19.1-fold (Table 1).

#### Variable region and D-loop

Shortening the variable region (ΔVr) decreased the catalytic efficiency by a factor 7.1, compared to wild-type *Pf*-apitRNA^Tyr^ (Table 1). However, insertion of the variable region of *Pf*-apitRNA^Ser^ (SerVr) reduced this effect to only a 2.7-fold reduction in activity, suggesting that our ΔVr deletion caused a change in tRNA structure rather than a direct impact on enzyme recognition. In *T*. *thermophilus* tRNA^Tyr^, A_20B_, located in the D-loop, interacts with U_47i_ in the variable domain, which provides a precise orientation of the variable region for its optimal recognition by TyrRS [35]. Since the *Pf*-apitRNA^Tyr^ sequence displays both A_20B_ and U_47i_, the same tertiary interaction could form (Fig 3A). Replacement of A_20B_ by U_20B_ in the *Pf*-apitRNA^Tyr^ D-loop should, therefore, eliminate this interaction. However, this mutant showed no loss in tyrosylation activity (1.6-fold) (Table 1). Finally, both the D-loop and the *Pf*-apitRNA^Tyr^ variable region were changed to the *Pf*-apitRNA^Ser^ (SerVr+D-l) sequences. In contrast to the long variable region in *Pf*-apitRNA^Tyr^, the long variable region of *Pf*-apitRNA^Ser^_GCU_ exhibits eight base pairs and potentially a specific tertiary interaction between G_45_-C_48a_ and U_20B_, determining its spatial orientation as in *T*. *thermophilus* [35] (S4 Fig). This replacement had no significant effect on tyrosylation efficiency (2.1-fold). Together these mutants demonstrate that, in the apicoplast, the presence of a long variable region plays a weak but significant role in tyrosylation identity (ΔVr, 7.1-fold), but neither the sequence nor the orientation of this long variable region is involved.

## Discussion

### Expression of *Pf*-apiTyrRS

*Plasmodium* aaRSs are longer than their homologs because they contain many peculiar, sequence-repetitive insertions [3]. Neither the synthesis nor the functions of these insertions are understood [33]; the presence of long single amino acid repeats often reduces the solubility of the recombinant protein, but they cannot be removed under penalty of obtaining an inactive protein (*e*.*g*, [47]). Insertions are more frequent and more extended in apicoplast than in cytosolic *Plasmodium* aaRSs. Moreover, it has been suggested that the translation of these additional sequences locally reduces the rate of ribosomes and could be used to regulate co-translational folding of proteins [6]. The presence of these insertions and the challenges they introduce may explain why apicoplast aaRSs are poorly studied despite their interest for the development of new anti-malarial drugs [13–15]. To date, only four apicoplast aaRSs from *P*. *falciparum* have been cloned, expressed, and characterized, namely lysyl- (LysRS) [48], glutamyl- (GluRS) [49], tryptophanyl- (TrpRS) [15], and the dual-targeted cysteinyl-tRNA (CysRS) synthetases [8]. CysRS and GluRS do not contain insertions, thus LysRS and TrpRS are the only insertion-containing apicoplast aaRSs that have been studied to date.

Translation is influenced by the choice of synonymous codons, which specify the same amino acid but differ in their decoding properties [50]. Thus, the primary structure of mRNA contains information that affects translation efficiency. The dominant model is that some codons or codon combinations reduce the decoding rate of ribosomes and thereby isolate the synthesis and folding of well-defined protein domains (*e*.*g*. [51–53]). The availability of tRNAs that decode synonymous codons, their requirement for wobble decoding, as well as interactions between adjacent codons play fundamental roles in this model. Codon usage and the number of tRNA genes in *Plasmodia* are very different from those of *E*. *coli* and thus make the expression of *Plasmodium* multidomain proteins challenging in the *E*. *coli* heterologous expression system. In this study, expression of recombinant *Pf*-apiTyrRS directly from the *Plasmodium* mRNA sequence was indeed ineffective. The optimization of synonymous codons involves the selection of optimal codons decoded only by abundant tRNAs in the expression host and thus the simultaneous minimization of rare codons [50,54]. This approach further reduced the solubility of the produced *Pf*-apiTyrRS. However, the use of harmonized codons, designed by the ANACONDA algorithm [21,22], increased the synthesis, solubility, and enzymatic activity of the purified recombinant protein. The main difference between both the Endo and the Opt genes compared to the Harm gene is the systematic replacement of leucine codons (TTA, TTG, CTG, CTT, and CTC) with the rarest leucine codon used in *E*. *coli* translation (CTA). As the ribosome slows when it encounters rare codons it may help the protein to fold appropriately, thereby increasing the yield of soluble proteins. Here, the Harm gene, containing a combination of fast and slow codons, facilitates co-translational folding and thus the production of a biologically active *Pf*-apiTyrRS. This result suggests that such an approach could be used to overcome the difficulties encountered when expressing *Plasmodium* multi-domains proteins.

### Evolution of tyrosine identity

Experimental work on tyrosylation systems from different species has established the evolution of the tyrosine identity set (Fig 2C). The A_73_ discriminator base and the G_34_ and U_35_ anticodon bases were determined as common identity elements in tRNA^Tyr^ of bacteria, archaea, eukarya, and mitochondria (summarized in [30]). In addition, the 1–72 base-pair at the end of the acceptor stem is critical to archaeal and eukaryal tyrosylation systems, whereas the long variable region is required only for the correct recognition of bacterial tRNAs (Fig 2C).

From our mutational analysis, the *Plasmodium* apicoplast tyrosylation system retains only one moderate (G_34_) and two weak (U_35_ and the long variable region) identity elements to ensure specific aminoacylation. Unlike other tyrosylation systems, this identity set does not include residues in the tRNA acceptor arm. Indeed, despite its conservation in all *Plasmodium* apitRNA^Tyr^, the first A_1_-U_72_ base pair was not involved in tyrosylation, a situation already observed for the human mitochondrial tyrosine system [44]; and the A_73_ discriminator base, common to all tyrosylation identity sets, does not influence tyrosylation in the *Plasmodium* apicoplast. Besides, neither the structure nor the orientation of the variable region is sufficient to prevent apicoplast tyrosylation. In the *Plasmodium* apicoplast system, insertion of the tRNA^Ser^ variable region into *Pf*-apitRNA^Tyr^ (mutants SerVr and SerVr+D-l) does not significantly reduce its recognition by *Pf*-apiTyrRS (2.7 and 2.1-fold, Table 1), while swapping the sequence of the variable region of *E*. *coli* tRNA^Tyr^ with that of *E*. *coli* tRNA^Ser^ decreases tyrosylation by more than 300-fold [40].

The only critical effect in the anticodon was obtained when G_34_ was mutated, which led to a loss in efficiency of only 36-fold; an unprecedented situation amongst tyrosylation systems. However, the identity of tRNAs is not only dictated by the presence of sets of positive identity elements allowing recognition by cognate synthetases, but also by negative signals that prevent the interaction of tRNAs with non-cognate synthetases. This scenario could play an important role in the *Plasmodium* apicoplast. Of the 27 tRNA gene sequences encoded by this genome, four contain a G_34_T_35_ sequence (*Pf*-apitRNA^Tyr^_GTA_, tRNA^Asn^_GTT_, tRNA^Asp^_GTC_, tRNA^His^_GTG_) and two contains a G_34_ and a long variable region (*Pf*-apitRNA^Tyr^_GTA_ and tRNA^Ser^_GCT_) (S4 Fig). Thus, the non-cognate asparagine, aspartate, histidine, and serine tRNAs must display features prohibiting recognition and tyrosylation by *Pf*-apiTyrRS. The transcription method used in the present study yields tRNAs lacking modified nucleotides, which may be a disadvantage if post-transcriptional modifications of native tRNAs play such a negative role in identity [17]. We can only predict that some modifications may be present in the apicoplast when the modification enzymes have been annoted in the *Plasmodium* genome [20]. For example, three putative queuine tRNA-ribosyltransferase are found in EupathDB, one of which (PF3D7_1242200) is predicted to be targeted to the apicoplast. Queuosine and its derivatives are found in bacterial and eukaryal tRNAs with a G34 [55], and guarantee fidelity and efficiency of translation [56]. The presence of this modifying enzyme in the apicoplast suggests that local tyrosine, histidine, aspartate, and asparagine tRNAs can be modified at position 34. However, nothing is known about post-transcriptional modifications of *Plasmodium* apicoplast tRNAs and if idiosyncratic modification patterns can control aminoacylation specificities.

We propose that the high A/T content of the *Plasmodium* apicoplast genome significantly reduces the potential for identity nucleotide combinations in apicoplast tRNAs. In the specific case of *Pf*-apitRNA^Tyr^, this led to the conservation of a minimal identity set with only three weak identity features positively recognized by the *Pf*-apiTyrRS. It is reasonable to ask whether these elements are sufficient to drive tyrosylation *in vivo* efficiently. Tyrosylation specificity could be mainly maintained by the presence of negative determinants (sequence/structural features or post-transcriptional modifications), which prevent mischarging of other *Pf*-apitRNAs by *Pf*-apiTyrRS.

## Supporting information

S1 Fig Multiple sequence alignments of eigth *Plasmodium* apiTyrRSs and comparison with the human mitochondrial TyrRS. Protein sequences are from EupathDB: *P*. *falciparum_3D7* (PF3D7_1117500), *P*. *reichenowi_CDC* (PRCDC_1115900), *P*. *vivax_P01* (PVP01_0918100), *P*. *knowlesi_strain_H* (PKNH_0915200), *P*. *gallinaceum_8A* (PGAL8A_00344100), *P*. *yoelii_yoelii_YM* (PYYM_0931900), *P*. *chabaudi_chabaudi* (PCHAS_0913800) and *P*. *berghei_ANKA* (PBANKA_0930500). The color code follows that of Fig 1A: residues belonging to the catalytic domain are in black with class I signature motifs highlighted in red; residues from the anticodon-binding domain are in green; the S4-like domain is in grey; and the two *Plasmodium*-specific insertions are in yellow. The starting position of recombinant *Pf*-apiTyrRS_25-561_ is indicated in cyan. Alignments were performed with Tcoffee [23] and predicted β-sheets and α-helices of *Pf*-apiTyrRS predicted by the PredictProtein software [20] are indicated with green arrows and red rectangles, respectively.
(DOCX)

S2 Fig DNA sequences encoding *Pf*-apiTyrRS. Alignment of nucleotide sequences corresponding to the endogeneous (Endo), optimized (Opt) and harmonized (Harm) gene sequences encoding *Pf*-apiTyrRS. The amino acid sequence of the protein is in bold. All leucine (L) codons are highlighted: red indicates codons whose usage in *E*. *coli* is higher than 1% (TTG, TTA, CTG, CTT and CTC) and in green for the only rare leucine codon (CTA, 0.385%) [26].
(DOCX)

S3 Fig Purification of *Pf*-apiTyrRSs and ATP/PPi exchange assays. (A) SDS-PAGE of purified *Pf*-apiTyrRS Endo, Opt and Harm (55 and 72 kDa protein molecular weigth markers are indicated). (B) Comparison of the purification yields of *Pf*-apiTyrRS expressed from Endo, Opt and Harm genes (relative to Endo); two independent purifications are shown (dark and light grey). (C) Gel filtration profile of *Pf*-apiTyrRS_25-561_ Harm (blue); gel filtration standards (grey) correspond to (1) thyroglobin (670 kDa), (2) bovine γ-globine (158 kDa), (3) chicken ovalbumine (44 kDa), (4) equine myoglobine (17 kDa) and (5) vitamine B-12 (1.35 kDa). (D) ATP/PPi exchange assays. The experiments were performed according to [57], in the presence of tyrosine (2 mM) and radiolabeled [^32^P]Ppi (20 cpm/pmol, this high specific activity was used to detect low exchange activities) and 0.8 μM *Pf*-apiTyrRS_25-561_ (squares) or the *Pf*-apiTyrRS_25-460_ deprived of its S4-like domain (spheres). A negative control with *Pf*-apiTyrRS_25-561_ without tyrosine was done in parallel (triangles).
(TIF)

S4 Fig Sequences of *P*. *falciparum* apicoplast tRNAs. Structural alignment of *P*. *falciparum* apitRNA genes [58] with accession numbers. The anticodon sequences are indicated in red. The domains of the cloverleaf structure are indicated at the top: Acc-s: acceptor-stem; D-s: D-stem; D-l: D-loop; Ac-s: anticodon-stem; Ac-l: anticodon-loop; T-s: T-stem; and T-l: T-loop. Note the presence of introns in the tRNA^Leu^_TAA_ and potentially in tRNA^Met^_CAT_-2 gene sequences. On the basis of their sequences, we could not assign initiator versus elongator functions to the two tRNA^Met^ isoacceptors.
(DOCX)

S1 Table Raw data values obtained in aminoacylation experiments. (A) Data used in Fig 1C correspond to the percentage of aminoacylated tRNAs after 5, 10 and 15 min incubation, in three independent experiments in the presence of Endo, Opt and Harm *Pf*-apiTyrRS. Corresponding means and errors (S.D.) are given and the p-values (t-test) were calculated for the Harm *Pf*-apiTyrRS compared to Endo (p-value(Endo)) or Opt (p-value(Opt)) *Pf*-apiTyrRSs. Significant p-values at p<0.05 are indicated in red. (B, C) Data corresponds to the kinetic parameters presented in Table 1. The individual K_m_ and k_cat_ values used to calculate means, errors (S.D.) and p-values are indicated. These apparent kinetic parameters were determined from Lineweaver-Burk plots. Significant p-values at p<0.05 (t-test) are shown in red and Loss values correspond to losses of catalytic efficiency relative to wild-type *Pf*-apitRNA^Tyr^.
(PDF)
