# Supplementary material for: Plasmodium apicoplast tyrosyl-tRNA synthetase recognizes an unusual, simplified identity set in cognate tRNATyr
Source: PLoS One. 2018 Dec 28;13(12):e0209805. doi: 10.1371/journal.pone.0209805 (PMC6310243; doi:10.1371/journal.pone.0209805)
Supplement: S1 Fig — Protein sequences are from EupathDB: P. falciparum_3D7 (PF3D7_1117500), P. reichenowi_CDC (PRCDC_1115900), P. vivax_P01 (PVP01_0918100), P. knowlesi_strain_H (PKNH_0915200), P. gallinaceum_8A (PGAL8A_00344100), P. yoelii_yoelii_YM (PYYM_0931900), P. chabaudi_chabaudi (PCHAS_0913800) and P. berghei_ANKA (PBANKA_0930500). The color code follows that of Fig 1A: residues belonging to the catalytic domain are in black with class I signature motifs highlighted in red; residues from the anticodon-binding domain are in green; the S4-like domain is in grey; and the two Plasmodium-specific insertions are in yellow. The starting position of recombinant Pf-apiTyrRS25-561 is indicated in cyan. Alignments were performed with Tcoffee [23] and predicted β-sheets and α-helices of Pf-apiTyrRS predicted by the PredictProtein software [20] are indicated with green arrows and red rectangles, respectively. (DOCX) [file pone.0209805.s001.docx]

*Hs-mito*  MAAPILRSFSWGRWSGTLNLSVLLPLGLRKAHSGA--------------------------------QGLLAAQKARGLFKDFFPETGTKIELPELFDRGTASFPQTIYCGFDPTADSLHVGHLLALLGLFHLQRAGHNVIALVGGATARL

              *.**:  .                               .  *  * *:: .    . .  * .  :.   .  :::*.*:* *.. **:*:*:.*: *  *:. . :** *:*.:*:::

----------------------------------------25-----------------------------------------------------------------------------98-101--------------------------

*P.falciparum* ----------------MIILKVLFIYNGLIQIIRCYKKFA**P**HRLNNII-----HENNKNNIGTYDIKSKALKKLYERKLIHYVSDIRNIDKILYHNENEKEKKNRKSVYAGIDLTCKYLHLGNLVPLITLDILRNHNTDVIILLGNSTTQI
*P.reichenowi*----------------MIILKVLFIYNGLIQIIRCYKKFAPHRLNNII-----HENNKNDIGTYDIKSKALKKLYERKLIHYVSDIRNIDKILYHNENEKEKKNRKSVYAGIDLTCKYLHLGNLVPLITLDILRNHNTDVIILLGSSTTQI
*P.vivax* --------------MVGWSSCVLLLLTHAA-QTECYKLHPHQSLSSSK-----ASLGESQTERHEIKSKALKKLFERKIIHYVSDVKAVDAMLHSNETEPKREKRKAVYLGIDVNCKYLHLGNLVQLITLDILRNHKTDVVVLLGGSTTMI
*P.knowlesi* --------------MMVGSSLYLLLLLAHAVQTQCYKFQPHQCLSSSK-----PPFGDSQTKKHEIKSKALKKLFERKLIHYVSDIEGVDNILYHNETESKKEKRKSVYLGIDVNCKYLHLGNLLQLMILDILRNHNTDIVVLLGGSTTKI
*P.gallinaceum* --------------MIKTYFILLKIILIFNSFIKCYKKCIPQFLTS--------RIEDNRIGTYEIKSKALKKLYERKIIHYVSDIKSIDEILYKNENEEERKNRKSVYVGIDLNCKYLHLGNLIPLITLDILRNYNTDIIILLGSSTTKI

*P.yoelii* ----------MMKITKLLILGLGILLFEKNVGIQCYKRSSIIKCSHTNSQNINKSIVKEKICEYKIKSNALKKLYERKLINYISDLKNVDQILYDNENE--NENKKSVYLGIDLNCKYLHIGSLVQLKTLEILRNYKTDVIILLGNSTTKI
*P.chabaudi* ----------MMKITKLLLLGLGILLFDNNVEVRCYKRSFILQCNNGNNQHINKNIIKDNTCEYKIKSNALKKLYERKLINYTSDIKGIDKILYENEQE--NENKKSVYVGIDLNCKYLHIGSLIPLTVLEILRNYKTDVIILLGNSTTKI

*P.berghei* ----------MMKINKLLILGLGILLFNKNVEIQCYKRSSIIKCNNANSQNINKSIVKEKICEYKIKSNALKKLYERKLINYISDLKNIDKILYENEHE--NEKKKSVYIGIDLNCKYLHIGSLTQLKTLEILRSYKTDVIILLGNSTTQI

                                         .***       .            ..    :.***:*****:***:*:* **:. :* :*: ** *  .:::*:** ***:.*:***:*.*: *  *:****::**:::***.*** *

*Hs-mito*  GDPSGRTKEREALETERVRANARALRLGLEALAANH---------------------------------------QQLFTDGRSWGSFTVLDNSAWYQKQHLVDFLAAVGGHFRMGTLLSRQSVQLRLKSPEGMSLAEFFYQVLQAYDFYY
 **** :. **:    : :  * . :*  :  *  :           :::: :. . **: :*.** **:* :::***   * :* :..** ::..  ::*  ..::* :: * .**::** :

------------------------------------167-----------------------------------186--------------------------------------------------------------------------

*P.falciparum* GDPSFQKVERQKTLEKDILENEENIRRTIIELFLQREICEED-M------------------NELIK-KSNIERDKEFIYESDNKGSLIILKNSLWYDKMNIIDFLK-YGEYFSINKLLRKECFLNKIK--KNLTLKDLNYITLQSFDFLH

*P.reichenowi*GDPSFQKVERQKTLEKDIIENEENIRRTIIELFLQREICEED-M------------------NELIK-KSNIERDKEFIYESDNKGSLIILKNSLWYDKMNIIDFLK-YGEHFSINKLLRKECFLNKFK--KNLTLKDLNYITLQSFDFLH
*P.vivax* GDPSFQQADRKRPFHEEIRENEESIRSTIVRLLLRGGTPNMS-T------------------SP----------LGDFSIDEPNKGSLTIVNNREWYDQMDLVDFLT-HGQHFSLHRILKKDCFKAKLKG-NNLTLKDLNYLILQSYDFVH
*P.knowlesi* GDPSFQQAERRKPIHEEISENEESIKGNIIRLLLRGEELNVG-T------------------SPGGKYDMGGNTQDEFSIHRPNKGSLTIVNNRQWYKQMDLADFLI-HGQYFALHKILKKECFKDKLKG-NNLTLKDLNYLILQSYDFVH
*P.gallinaceum* GDPSFQNTKRKKTVDEEICENEKNIRTNIINLFLQNELSEKH-L------------------NEIIE-KSNILSNKEFIYESKKKGSLIILKNNFWYDNINIIDFLK-YGEYFSVNKLLRKECLSSKLK--KNLTLKDLNYITLQSFDFLY

*P.yoelii* GDPSFQLNERKETLNDEICENEKNIKKSIIDFILNNDNKDYINEKDLPMKKLCGINLNKISFQEICR-KSDLLSKDKLIYKCSGKGSLRILKNNIWYDKMNIIDFLK-HGGHFGINKLLRKESVIKKYQN-KKLTLKDLNYISLQAYDFLY
*P.chabaudi* GDPSFQTNERKQTLNEEIYQNEKSIKETIIDYILNNDNSNFN-ATDFPMRQLCGMNLNQMSFQDICK-KTDLLSKDEIIYECNGKGSLRILKNNGWYDKMSVIDFLK-YGEHFGIHKLLRKDSVIKKYQN-KNLTLKDLNYISLQSYDFLY
*P.berghei* GDPSFQLNERKETLNAEICQNEQSIKESIIDFILNNDNSDFN-EKDLHMKELCGINLNQISFQDICR-KTDLLSKDEIIYKCSGKGNLRILKNNIWYDKMNVIDFLK-YGEHFGINKLLRKESVIKKYQN-KNLTLKDLNYISLQSYDFLH

           ******  .*:.....:* :**:.*:  *:  :*.                      .:  .   **.* *::*  **.::.: ***  :* :* ::::*:*:..  * :  .:********: **::**::

*Hs-mito*  LFQRYGCRVQLGGSDQLGNIMSGYEFINKLTGEDVFGITVPLITSTTGAKLGKSAG-----NAVWLNRDKTSPFELYQFFVRQPDDSVERYLKLFTFLPLPEIDHIMQLHVK---------------------------------------
 ** : :::*:***** *** ** *: : ::. :::*:*. *:. ... * .**        .:*:::: .**: :::*: .  *:.*: *:.::* * :   :.*  ::

-------------------------------------------------308-312--------------328-------------------------------------368--------------------------------------

*P.falciparum-* LFNKFKTCIQIGGSDQWGNIQSGIELAQYISNTQLYGLTTNLLVYKNNIKYSKSQFNENKRLPIWIDKNYNSPYLFWNFLRNVEDQKVQSYIDMLTNLKININQEIETVYNPM--DTNLNEILDETLDDSNKKKKNNDNNNNNNNVKDINI
*P.reichenowi*LFNKFKTCIQIGGSDQWGNIQSGIELAQYISNTQLYGLTTNLLVYKNNIKYSKSQFNENKRLPIWIDKNYNSPYLFWNFLRNVEDQKVQSYIDMLTNLKININQEIETLYNPM--DRNLNETLDDSKKKNDDDD--------NNNMKDIDI
*P.vivax* LYKRYRCCIQIGGSDQWGNIQSGIELCQHLCNEQLYGLTTNLLLHRNDTKYSKSLFEQNRKMPIWIDRDYTPPFLFWNFLRNVDDQQVDSYLCMLTGLEVQGGDPPSEPTPS---------------------------------------
*P.knowlesi* LYKKYRCFIQIGGSDQWGNIQSGIELCQHLHNTQLYGLTSNLLLHKNNTKYSKSLFEQNRKIPIWIDKEYTPPFLFWNFLRNIDDQQVDSYISMLTDLDVQSRNPSSDIT-----------------------------------------
*P.gallinaceum* LFQNFQTFIQIGGSDQWGNIQSGIELCQNIFKTQLYGFTTNLLLHKNNVKYSKSLFNENRKLPIWIDIHYNPPYLFWNFLRNIEDQKVQSYIDMLTDINIDINEKISNYSDSNYREKNENEKIDNT-------------------------

*P.yoelii* LFKKYNTYIQIGGSDQWGNIQSGIEFCQNIYNKQLYGLTTNLLLYKNNIKFSKSLFHENKKLPIWIDTEYTSPYLFWNFFRNVEDQKVQSYIDMLTNLNINLDEELKNEVLKN--EELKNEVLKNEELKNEELKNEELKNEELKNEELKNE
*P.chabaudi* LYKKYKTYIQIGGSDQWGNIQSGIEFCQNIYNQQLYGLTTNLLLYKNNMKFSKSLFYENKKLPIWINKEYTPPYLFWNFLRNVEDQKVQSYIDMLTNLNINLDEELKKSDHE---------------------------------------
*P.berghei* LFKKYKTYIQIGGSDQWGNIQSGIELCQNIYNQQLYGLTTNLLLYKNNIKFSKSLFQENKKLPIWIDKEYTSPYLFWNFLRNVEDQKVQSYIDMLTNLNINLDEELKNSN-----------------------------------------

              *::. :. *****************:.* : : ****:*:***:::*: *:*** * :.:::****: .*..*:*****:**::**:*::*: ***.:.:

*Hs-mito*  -----------------EPERRGPQKRLAAEVTKLVHGREGLDSAKRCTQALYHSSIDALEVMSDQELKELFKEAPFSEFFLDPGTSVLDTCRKANAIPDGPRGYRMITEGGVSINHQQVTNPESVLIVGQ-HI—LK---NGLSLLKIGKR
      * : . .:*:*: .**. :.*.. :.. :: .:.* :..:. :: :.* ::          *.: .   .: *  :* :  . . .. . *::. : :*.  :.:.:  * :.: * *   * ::*::***
 ----------------424----------------------------------460-----------------------------------------------------------------------------------------------

*P.falciparum-* TTEHNNYSNDISLEKSYEEKINQAKKQLSDSVTSYIFGEHTVKKIHKMKDVLKNNEFHKINNIDDIKVFP--YVEITMEHINKKQINISDLLKKFDIASTNKEAKEKISQNCIYLNELLINDSKYSLNINN-FIKLH--NNYYAILRLGKR
*P.reichenowi*TSQHNNYSNDISLEKSYEEKINQAKKQLSDSVTSYIFGEHTVKKIHKMKDVLKNNEFHKINNIDDIKVFP--YVEITMEHINKKQLNISDLLKKFHIASTNKEAKEKISQNCIYLNELLINDSKYQLNINN-FIKLH--NNYYAILRLGKR
*P.vivax* --------PSEGAEATGDAHINRKKEKLADAVTAFIYGEETVRTIHTLSRLLKEDRFAVVDRVDQLKVFP--FILIRRSDLHNKQISIVHILRRFHVAATNKEAKEKLAQRCIYLNRRLVEDAKYRLSLASSFVRAK-DGSYYAVLGLGRK
*P.knowlesi* ---------SGGEDYHDDAQINRAKEKLADSVTTFVYGAETVCTIHTLNRLLKEDEFSVIDRVDQLKVFP--FIVINKNDIHKNDISIVHILRKFEVASTNKEAKEKLAQRCIYLNRQLIDNAKYHLSMPSSFVKAK-DGNYYAILGLGRK
*P.gallinaceum* ----TINKNTINLEKIYEEEINKAKKKLSDSVTSYIYGKEVVKKIHKMNKMIKDEQTYEIENIEDLKIFP--FLEISKDDLNEKKIDILQILKKFEIAETNKEAKEKINQKCIYLNKDLITNPKLQLSISN-FLQIH--NNYYAILRLGKK

*P.yoelii* E--LKNENEKNGIASRYDDIINSAKKKMADHITNTIYGKSLVNKIHKINKIIKNNKFNEIENLQELKILP--YNQININLIKQNKINITDILKSFQIAMTNKQAKEKINQNCIYINTKLITDPKYILNIMD-FLKTKNDNNYYALLRLGKK
*P.chabaudi* -------SSKNRNISSYDNIINSAKKQMADHITNTIYGKSVINKIHKINNIIKNNQFNEIENLQELKILP--YNQININSIKQNEINITDVLKFFQIAMTNKEAKEKINQNCIYINTKLITDPKYVLTITD-FLKTKDHNYYYALLRLGKK
*P.berghei* -----NENPKNIIASSYDNIINSVKKKMADHITNTIYGKCVVDKIHKINKIIKNNKFNEIENLQELKILP--YNQININLIKQNKINITDVLKLFQIAMTNKEAKEKINQNCIYINTKLITNPKYILNIMD-FLKTKNDNYYYVLLRLGKK

                     :  **  *::::* :*  ::*   :  **.:. ::*::.   ::.::::*::* :  *  . ::::.:.* .:*: *.:* ***:****: *.***:*  *: :.*  *.: . *:: :  . ***:* **::

*Hs-mito*  NFYIIKWLQL
 . * *     
 -------561

*P.falciparum-* TSYSIIIK----------
*P.reichenowi*TSYSIIIK----------
*P.vivax* TYYSVIVQ----------
*P.knowlesi* TYYSIIVQ----------
*P.gallinaceum* TFYSIIVK----------

*P.yoelii* SSYSIIAKYDKKETLNND
*P.chabaudi* SAYSIIAKPDEENSN—Q
*P.berghei* SSYSIIAKSD--------

           : **:* :
