# Supplementary material for: Plasmodium apicoplast tyrosyl-tRNA synthetase recognizes an unusual, simplified identity set in cognate tRNATyr
Source: PLoS One. 2018 Dec 28;13(12):e0209805. doi: 10.1371/journal.pone.0209805 (PMC6310243; doi:10.1371/journal.pone.0209805)
Supplement: S2 Fig — Alignment of nucleotide sequences corresponding to the endogeneous (Endo), optimized (Opt) and harmonized (Harm) gene sequences encoding Pf-apiTyrRS. The amino acid sequence of the protein is in bold. All leucine (L) codons are highlighted: red indicates codons whose usage in E. coli is higher than 1% (TTG, TTA, CTG, CTT and CTC) and in green for the only rare leucine codon (CTA, 0.385%) [26]. (DOCX) [file pone.0209805.s002.docx]

**M P H R L N N I I H E N N K N N I G T Y D I K S K A L K K L Y E R K L I H Y V S D I R N I D K I L Y H N E N E K E K K N**

Endo ATGCCTCATAGATTGAATAACATAATTCACGAGAATAATAAAAATAACATAGGTACCTATGATATTAAATCGAAAGCTTTAAAAAAATTATATGAAAGGAAATTGATTCATTATGTAAGTGATATAAGAAATATTGATAAGATATTATATCACAATGAAAATGAGAAAGAAAAGAAAAAT

Opt ATGCCGCATCGCCTGAATAACATTATTCACGAGAATAACAAGAATAACATTGGTACCTATGATATTAAAAGTAAAGCGCTGAAAAAACTGTACGAACGTAAACTGATTCATTACGTTAGCGACATCCGCAACATCGACAAAATCCTGTATCACAACGAGAATGAGAAAGAAAAGAAAAAC

Harm ATGCCCCATAGGCTAAACAATATCATCCACGAGAACAATAAGAACAATATCGGCACATATGATATCAAAAGCAAAGCTCTAAAAAAACTATACGAACGGAAACTAATCCATTACGTGTCAGACATAAGGAATATAGACAAAATACTATATCACAATGAGAACGAGAAAGAAAAGAAAAAT

***** *** * * ** ** ** ** ******** ** ** ** ** ** ** ** ******** *** ***** * ****** * ** *** * *** * ** ***** ** ** ** * ** ** ** ** ** * ******** ** ** *****************

**R K S V Y A G I D L T C K Y L H L G N L V P L I T L D I L R N H N T D V I I L L G N S T T Q I G D P S F Q K V E R Q K T**

Endo AGAAAAAGTGTTTATGCAGGTATTGATTTAACATGCAAATATTTGCATTTAGGTAATTTAGTACCTTTGATTACTTTGGATATATTACGTAATCATAATACAGATGTTATTATATTATTAGGAAATAGTACCACACAAATAGGAGACCCTTCATTTCAAAAAGTAGAAAGACAAAAAACG

Opt CGTAAAAGCGTATACGCTGGCATTGATCTGACCTGTAAATACCTGCACCTGGGCAACCTGGTCCCACTGATCACCCTGGACATCCTGCGCAACCACAACACGGATGTGATTATCCTGCTGGGAAACAGTACCACGCAGATTGGTGATCCAAGCTTTCAAAAAGTGGAACGTCAGAAAACC

Harm CGGAAATCAGTGTACGCGGGAATCGATCTAACATGCAAATACCTACACCTAGGAAATCTAGTACCGCTAATAACACTAGACATACTAAGGAATCACAATACAGATGTAATCATACTACTAGGCAATAGCACAACACAAATCGGCGATCCGTCATTTCAGAAAGTAGAACGGCAAAAAACA

* *** ** ** ** ** ** *** * ** ** ***** * ** * ** ** * ** ** * ** ** * ** ** * * ** ** ** ** ***** ** ** * * ** ** ** ** ** ** ** ** ** ** ***** ***** *** * ** *****

**L E K D I L E N E E N I R R T I I E L F L Q R E I C E E D M N E L I K K S N I E R D K E F I Y E S D N K G S L I I L K N**

Endo TTGGAGAAAGATATATTAGAGAATGAAGAAAATATTAGAAGAACAATAATTGAATTATTTCTTCAAAGAGAAATATGTGAAGAAGATATGAATGAATTAATTAAAAAAAGTAATATAGAAAGGGATAAAGAATTTATATATGAATCAGATAATAAAGGTTCTTTAATTATATTAAAAAAT

Opt CTGGAAAAAGACATCCTGGAAAACGAGGAAAACATCCGCCGCACCATCATTGAACTGTTCCTGCAACGTGAAATCTGCGAAGAAGATATGAACGAACTGATTAAAAAGTCCAATATCGAACGTGATAAAGAGTTCATTTACGAAAGTGATAACAAAGGTTCGCTGATTATTCTGAAGAAC

Harm CTAGAAAAAGACATACTAGAAAATGAGGAAAATATAAGGAGGACAATAATCGAACTATTCCTACAGCGGGAAATATGTGAAGAAGATATGAATGAACTAATCAAAAAGTCAAACATAGAACGGGATAAAGAGTTCATCTACGAAAGCGATAATAAAGGCTCACTAATCATCCTAAAGAAT

* ** ***** ** * ** ** ** ***** ** * * ** ** ** *** * ** ** ** * ***** ** ************** *** * ** ***** ** ** *** * ******** ** ** ** *** ***** ***** ** * ** ** * ** **

**S L W Y D K M N I I D F L K Y G E Y F S I N K L L R K E C F L N K I K K N L T L K D L N Y I T L Q S F D F L H L F N K F**

Endo AGTTTATGGTATGATAAAATGAATATAATTGATTTTTTAAAATATGGAGAATATTTTTCTATTAATAAATTATTAAGAAAAGAATGTTTTCTTAATAAAATTAAAAAAAACCTTACATTGAAAGATTTAAATTATATAACATTACAATCATTTGATTTTTTACATTTGTTTAATAAATTT

Opt TCACTGTGGTATGACAAGATGAATATTATCGATTTTCTGAAATATGGCGAATACTTTAGCATTAACAAACTGCTGCGCAAAGAATGCTTCCTGAACAAAATTAAGAAAAACCTGACCCTGAAGGATCTGAATTATATCACACTGCAAAGTTTCGACTTCCTGCATCTGTTTAATAAATTT

Harm AGCCTATGGTATGACAAGATGAACATCATAGATTTTCTAAAATATGGAGAATACTTTTCAATCAATAAACTACTAAGGAAAGAATGTTTCCTAAATAAAATCAAGAAAAATCTAACACTAAAGGATCTAAACTATATAACCCTACAGAGCTTCGACTTCCTACATCTATTTAACAAATTT

* ******** ** ***** ** ** ****** * ******** ***** *** ** ** *** * * * ******** ** ** ** ***** ** ***** ** ** * ** *** * ** ***** ** * ** ** ** ** * *** * ***** ******

**K T C I Q I G G S D Q W G N I Q S G I E L A Q Y I S N T Q L Y G L T T N L L V Y K N N I K Y S K S Q F N E N K R L P I W**

Endo AAAACCTGTATACAAATAGGAGGATCAGATCAATGGGGTAATATACAATCAGGAATAGAACTTGCTCAGTATATATCTAATACTCAATTATATGGTTTAACTACTAATTTATTAGTATATAAAAATAATATAAAATATAGTAAGTCTCAATTTAATGAAAATAAAAGGTTACCTATATGG

Opt AAAACCTGCATCCAAATCGGTGGGAGCGATCAATGGGGTAACATCCAGTCGGGCATTGAACTGGCGCAATATATTTCTAATACCCAGCTGTACGGCCTGACGACAAACCTGCTGGTGTACAAAAATAACATCAAGTACAGTAAATCGCAGTTCAACGAAAATAAACGTCTGCCAATTTGG

Harm AAAACATGTATACAGATAGGCGGATCAGATCAGTGGGGCAATATACAATCAGGAATCGAACTAGCTCAGTATATCAGCAACACACAACTATACGGACTAACAACCAATCTACTAGTATACAAAAACAATATAAAGTACAGCAAATCACAATTCAATGAAAACAAACGGCTACCGATCTGG

***** ** ** ** ** ** ** ***** ***** ** ** ** ** ** ** ***** ** ** ***** ** ** ** * ** ** * ** ** ** * * ** ** ***** ** ** ** ** ** ** ** ** ** ** ***** *** * * ** ** ***

**I D K N Y N S P Y L F W N F L R N V E D Q K V Q S Y I D M L T N L K I N I N Q E I E T V Y N P M D T N L N E I L D E T L**

Endo ATAGATAAAAATTATAACTCTCCATATTTATTCTGGAATTTTTTAAGAAATGTAGAAGATCAAAAGGTTCAATCATATATTGATATGTTAACTAATTTAAAAATAAATATAAATCAAGAAATTGAAACAGTATATAATCCAATGGATACAAACTTAAATGAGATTTTGGATGAGACTTTG

Opt ATTGATAAAAACTATAATAGCCCTTATCTGTTTTGGAACTTTCTGCGCAATGTCGAGGATCAAAAAGTGCAGAGCTACATTGATATGCTGACTAACCTGAAGATTAACATCAACCAGGAAATTGAAACCGTCTATAATCCGATGGATACTAATCTGAATGAAATCCTGGATGAAACCCTG

Harm ATCGATAAAAATTATAACTCACCGTATCTATTTTGGAATTTTCTAAGGAACGTAGAGGATCAGAAAGTACAATCATACATCGATATGCTAACGAATCTAAAGATCAATATAAATCAAGAAATCGAAACAGTATATAACCCCATGGATACGAACCTAAACGAAATACTAGATGAAACACTA

** ******** ***** ** *** * ** ***** *** * * ** ** ** ***** ** ** ** ** ** ****** * ** ** * ** ** ** ** ** ** ***** ***** ** ***** ** ******** ** * ** ** ** * ***** ** *

**D D S N K K K K N N D N N N N N N N V K D I N I T T E H N N Y S N D I S L E K S Y E E K I N Q A K K Q L S D S V T S Y I**

Endo GATGACTCTAATAAAAAAAAAAAAAATAATGATAATAATAATAATAATAATAATGTGAAAGACATAAATATTACTACTGAGCATAATAATTATAGTAATGATATATCATTGGAAAAAAGTTATGAAGAAAAAATTAACCAAGCTAAAAAACAACTATCAGATAGTGTTACCTCTTATATT

Opt GATGATTCCAATAAAAAGAAAAAGAACAACGACAACAACAACAACAATAATAATGTGAAGGACATCAACATTACCACCGAACATAACAATTATAGCAATGATATTTCTCTGGAGAAAAGCTATGAAGAAAAAATCAACCAAGCTAAAAAACAGCTGAGCGATTCTGTGACTAGCTACATC

Harm GATGATTCAAACAAAAAGAAAAAGAATAATGACAATAATAATAATAACAACAACGTAAAGGACATAAATATCACAACAGAACATAATAACTATTCAAACGATATCAGCCTAGAGAAATCATATGAAGAAAAAATAAATCAGGCGAAAAAACAACTATCAGATAGCGTAACGTCATACATA

***** ** ** ***** ***** ** ** ** ** ** ** ** ** ** ** ** ** ***** ** ** ** ** ** ***** ** *** ** ***** * ** *** ************** ** ** ** ******** ** *** ** ** ** **

**F G E H T V K K I H K M K D V L K N N E F H K I N N I D D I K V F P Y V E I T M E H I N K K Q I N I S D L L K K F D I A**

Endo TTTGGTGAACACACTGTTAAAAAAATACATAAAATGAAAGATGTTTTAAAGAATAATGAATTTCATAAAATTAATAATATTGATGATATAAAAGTATTTCCATATGTTGAAATAACAATGGAACATATAAATAAAAAGCAAATAAATATTTCAGATTTATTGAAGAAATTTGATATTGCA

Opt TTTGGGGAACATACAGTAAAGAAAATTCACAAAATGAAAGATGTTCTGAAAAATAACGAATTTCACAAAATTAACAACATTGATGATATCAAAGTCTTTCCTTATGTGGAAATTACCATGGAGCATATTAATAAAAAACAAATCAATATCTCGGATCTGCTGAAAAAATTTGACATTGCT

Harm TTTGGAGAACATACCGTGAAGAAAATCCACAAAATGAAAGATGTGCTAAAAAACAATGAATTTCACAAAATCAATAATATCGATGATATAAAAGTATTTCCGTATGTAGAAATCACAATGGAGCATATCAACAAAAAACAGATAAACATATCAGATCTACTAAAAAAATTTGACATCGCG

***** ***** ** ** ** ***** ** ************** * ** ** ** ******** ***** ** ** ** ******** ***** ***** ***** ***** ** ***** ***** ** ***** ** ** ** ** ** *** * * ** ******** ** **

**S T N K E A K E K I S Q N C I Y L N E L L I N D S K Y S L N I N N F I K L H N N Y Y A I L R L G K R T S Y S I I I K ***

Endo TCTACTAACAAAGAAGCTAAAGAAAAGATAAGTCAAAATTGTATATATTTAAATGAACTTCTTATAAATGATTCAAAATATTCATTGAATATAAATAACTTTATAAAGCTTCATAATAATTATTATGCAATCCTCAGGTTAGGCAAAAGAACAAGTTATTCTATAATAATAAAATGA

Opt AGCACAAACAAGGAAGCGAAAGAGAAAATCAGTCAGAATTGCATTTACCTGAATGAACTGCTGATTAATGATTCCAAATATAGCCTGAACATCAACAACTTTATCAAACTGCACAATAATTATTACGCCATTCTGCGTCTGGGCAAACGCACAATTAAACTGAATCAAATCAAATAA

Harm TCAACCAATAAGGAAGCTAAAGAGAAAATAAGCCAAAACTGTATCTACCTAAACGAACTACTAATCAACGATTCAAAATATTCACTAAATATAAATAATTTTATAAAACTACACAACAACTATTACGCTATCCTACGGCTAGGAAAAAGGACCAGTTATTCTATAATAATAAAATGA

** ** ** ***** ***** ** ** ** ** ** ** ** ** * ** ***** ** ** ** ***** ****** * ** ** ** ** ***** ** ** ** ** ** ***** ** ** ** * * ** *** * ** * * * * *** **** *
