# Supplementary figures and images for: Plasmodium apicoplast tyrosyl-tRNA synthetase recognizes an unusual, simplified identity set in cognate tRNATyr
Source: PLoS One. 2018 Dec 28;13(12):e0209805. doi: 10.1371/journal.pone.0209805 (PMC6310243; doi:10.1371/journal.pone.0209805)

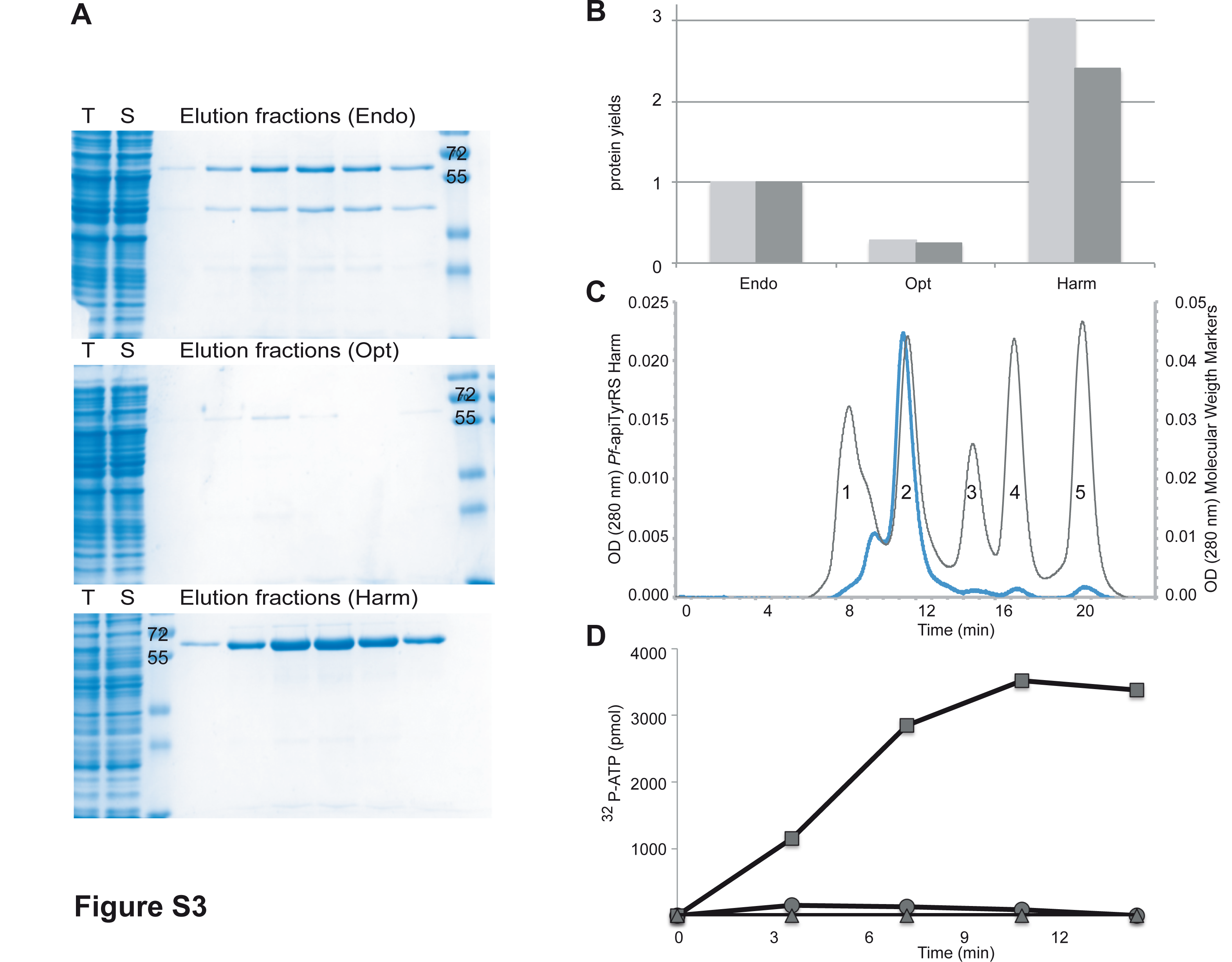

Supplement: S3 Fig — (A) SDS-PAGE of purified Pf-apiTyrRS Endo, Opt and Harm (55 and 72 kDa protein molecular weigth markers are indicated). (B) Comparison of the purification yields of Pf-apiTyrRS expressed from Endo, Opt and Harm genes (relative to Endo); two independent purifications are shown (dark and light grey). (C) Gel filtration profile of Pf-apiTyrRS25-561 Harm (blue); gel filtration standards (grey) correspond to (1) thyroglobin (670 kDa), (2) bovine γ-globine (158 kDa), (3) chicken ovalbumine (44 kDa), (4) equine myoglobine (17 kDa) and (5) vitamine B-12 (1.35 kDa). (D) ATP/PPi exchange assays. The experiments were performed according to [57], in the presence of tyrosine (2 mM) and radiolabeled [32P]Ppi (20 cpm/pmol, this high specific activity was used to detect low exchange activities) and 0.8 μM Pf-apiTyrRS25-561 (squares) or the Pf-apiTyrRS25-460 deprived of its S4-like domain (spheres). A negative control with Pf-apiTyrRS25-561 without tyrosine was done in parallel (triangles). (TIF) [file pone.0209805.s003.tif]
