# Supplementary material for: Plasmodium apicoplast tyrosyl-tRNA synthetase recognizes an unusual, simplified identity set in cognate tRNATyr
Source: PLoS One. 2018 Dec 28;13(12):e0209805. doi: 10.1371/journal.pone.0209805 (PMC6310243; doi:10.1371/journal.pone.0209805)
Supplement: S4 Fig — Structural alignment of P. falciparum apitRNA genes [58] with accession numbers. The anticodon sequences are indicated in red. The domains of the cloverleaf structure are indicated at the top: Acc-s: acceptor-stem; D-s: D-stem; D-l: D-loop; Ac-s: anticodon-stem; Ac-l: anticodon-loop; T-s: T-stem; and T-l: T-loop. Note the presence of introns in the tRNALeuTAA and potentially in tRNAMetCAT-2 gene sequences. On the basis of their sequences, we could not assign initiator versus elongator functions to the two tRNAMet isoacceptors. (DOCX) [file pone.0209805.s004.docx]

**5’ Acc-s D-s D-l D-s Ac-s Ac-l Ac-s variable region T-s T-l T-s Acc-s 3’**

>>>>>>> >>>> <<<< >>>>> <<<<< >>>>> <<<<< <<<<<<<

# AlaTGC (PF3D7_API05600) GGGAATATAGTTT....AATGGTA...AAATCTTATTTTTGCATAATAA.AGAT....................AGTAGTTCAATTCTACT.TATTTCC.A

# ArgACG (PF3D7_API05100) AAACTTGTAATTT....AATGGATA..AAATATATAAATACGAATTATA.AAAT....................AAAAGTTCAATTCTTTT.CAAGTTT.A

# ArgTCT (PF3D7_API05300) AAATCTATAATTT....AATGGATA..AAATAAAAACCTTCTAAGTTTT.ATAT....................GTAAGTTCAAATCTTAC.TAGATTT.A

# AsnGTT (PF3D7_API05500) TTCTTAATAGCTT....AGTGGTTA..AAGCATTCGGCTGTTAACCGAA.ATAC....................ACTAGTTCAATTCTAGT.TTAAGAA.G

# AspGTC (PF3D7_API00900) GAATTTGTTAGTT.TAATTAGGTAA..AAATATTATTTTGTCATAATAA.AGAAT...................ACGAGTTCAATTCTCGT.CAAATTC.G

# CysGCA (PF3D7_API00300) AATGATATAACTT....AATTGATA..AAGTAAATAATTGCAAATTATT.ATA.....................TTTCAGTTTGAATCTGA.ATATCAT.T

# GlnTTG (PF3D7_API03200) TAGAATATAACCA....AAAGGTTA..AGGTAATGAATTTTGATTTCAT.TAAT....................ATAGGTTCGAATCCTAT.TATTCTA.A

# GluTTC (PF3D7_API01100) ACTTTTATCGTTT....AAAGGTA...AGACATCTTTTTTTCAAGAAGA.AAA.....................TAGGAATTCGATTTTCC.TTAAAAG.T

# GlyACC (PF3D7_API03300) ATGAATATAATTT....AATGATA...AAATACAATTTTACCATAATTG.TTA.....................TAAGAGTTTGAATCTCT.TTATTCA.T

# GlyTCC (PF3D7_API03700) ACAAATATAGTTT...AATCGGTA...AAATATTAATTTTCCAAATTAA.TGAT....................ATGGATTCAATTTCCAT.TATTTGT.A

# HisGTG (PF3D7_API00200) ATAAATATAATCT....AATGGTTA..AGATGAAGAATTGTGGTTTCTT.TTAT....................ATGAGTTCAAATCTCTT.TATTTAT.C

# IleGAT (PF3D7_API05800) AGGTTTTTAGTTT....AATGGTTA..AAACATACTCTTGATAAGGGTA.AAAT....................TTTAGTTCAATTCTAAA.AAAACCT.A

# LeuTAG (PF3D7_API05400) ATGAATATGGCGA...AATAGGTAA..ACGCACTAAATTTAGATTTTAG.TTATTATAAT..............AAGAGTTCAAATCTCTT.TATTCAT.A

# LeuTAA (Pf3D7_API_v3:809..1,028-intron AGAGATATGGTGA..AATTTGGTAT..ACACAATGGACTTAAAATCCAT.TAACATTATTGTTGT.........AAGGGTTCAAATCCCTT.TATCTCT.A

# LysTTT (PF3D7_API01000) GAATTACTAGCTT...AATTGGTA...GAGTACTCGACTTTTAATCGAA.TGGTT...................CTGAGTTCAAATCTCAG.GTAGTTC.A

# MetCAT-1(PF3D7_API05000) AGCGAAATAGAGC...ATAAGGAAA..GTTCGTCGGATTCATGCTCCGA.AGGTA...................ATCGGTTCAATTCCGTT.TTTCGCT.T

# MetCAT-2(PF3D7_API00600)-intron ? ACATTTATAGCTA....AGTGGTCGA.AAGCAATGGACTCATAATTGAT.CATC....................AGTAGTTCGAATCTACT.TAAATGT.A

# PheGAA (PF3D7_API03100) GTCATAATAGCTC....AATGGTA...GAGCAATGGATTGAAGATCCAT.GTGTT...................ATCAGTTCAAATCTGAT.TTTTGAC.A

# ProTGG (PF3D7_API01200) CAGAATATAGTGT....AATGGTA...ACATATCTATTTTGGGAATAGA.AGAAT...................ATAGGTTCAAATCCTAT.TTTTCTG.A

# SerTGA (PF3D7_API03900) AGAGAAATGACAG....AGTGGTTTA.TTGTGTTTGATTTGAGATCAAA.AAAATATAAATATATTTC......ATGGGTTCAAATCCCAT.TTTCTCT.T

# SerGCT (PF3D7_API00800) AGAGAAATGACTG....AGAGGTTTA.TAGTTATAAATTGCTAATTTAT.TGTATATATAATAATATTATACC.AAGGGTTCGAATCCCTT.TTTCTCT.A

# ThrTGT (PF3D7_API04800) GCTAAAGTAGCTT...AATTGGTAA..AGCA.ACTGATTTGTAATCAGT.AGATT...................ATGAGTTCAAATCTCAC.CATTAGC.T

# ThrTGT (PF3D7_API04800) GCTAAAGTAGCTT...AATTGGTA...AAGCAACTGATTTGTAATCAGT.AGATT...................ATGAGTTCAAATCTCAC.CATTAGC.T

# TrpCCA (PF3D7_API03400) ATGTCTTTAATTT....AAAGTAA...AAATATAAATTTCCAAAATTTA.TAAT....................AAAGGTTCGAATCCTTT.AGGACAT.G

# TyrGTA (PF3D7_API00700) AAGTTAATGCCTG....AGTGGTTAA.AAGGAATGGACTGTAAATCCAT.TGATAATATATCTAC.........ATCAGTTCAAATCTGAT.TTAACTT.A

# ValTAC (PF3D7_API05200) AAGTAATTAACTT....AGAGGTA...AAGTTTCTGCTTTACATACAGA.AGACC...................ATTGGTTCGAATCCAAT.ATTACTT.A
