# Supplementary material for: Plasmodium apicoplast tyrosyl-tRNA synthetase recognizes an unusual, simplified identity set in cognate tRNATyr
Source: PLoS One. 2018 Dec 28;13(12):e0209805. doi: 10.1371/journal.pone.0209805 (PMC6310243; doi:10.1371/journal.pone.0209805)
Supplement: S1 Table — (A) Data used in Fig 1C correspond to the percentage of aminoacylated tRNAs after 5, 10 and 15 min incubation, in three independent experiments in the presence of Endo, Opt and Harm Pf-apiTyrRS. Corresponding means and errors (S.D.) are given and the p-values (t-test) were calculated for the Harm Pf-apiTyrRS compared to Endo (p-value(Endo)) or Opt (p-value(Opt)) Pf-apiTyrRSs. Significant p-values at p<0.05 are indicated in red. (B, C) Data corresponds to the kinetic parameters presented in Table 1. The individual Km and kcat values used to calculate means, errors (S.D.) and p-values are indicated. These apparent kinetic parameters were determined from Lineweaver-Burk plots. Significant p-values at p<0.05 (t-test) are shown in red and Loss values correspond to losses of catalytic efficiency relative to wild-type Pf-apitRNATyr. (PDF) [file pone.0209805.s005.pdf]

A

time (min)	Endo1	Endo2	Endo3	Means Endo	S.D. Endo		
	tRNA aminoacylation (%)						
5	0,0	3,0	0,8	1,3	1,3		
10	6,7	11,9	12,1	10,2	2,5		
15	22,5	20,8	17,9	20,4	1,9		
	**Opt1**	**Opt2**	**Opt3**	**Means Opt**	**S.D. Opt**		
5	2,0	1,1	3,7	2,2	1,1		
10	14,2	6,2	4,7	8,3	4,2		
15	22,0	13,1	16,2	17,1	3,7		
	**Harm1**	**Harm2**	**Harm3**	**Means Harm**	**S.D. Harm**	**p-value (Endo)**	**p-value (Opt)**
5	43,9	36,7	24,4	35,0	8,0	0,025	0,027
10	71,4	62,0	50,4	61,3	8,6	0,009	0,005
15	103,7	71,7	60,7	78,7	18,2	0,044	0,037

B

	Km1	Km2	Km3	Km4	kcat1	kcat2	kcat3	kcat4	Means (Km)	S.D. (Km)	Means (kcat)	S.D. (kcat)
E. coli $tRNA^{Tyr}$	0,97	0,96	0,6	1,07	8,1	7,6	13,8	18,3	0,9	0,21	12,0	5,08
Pf-$apitRNA^{Tyr}$	0,48	0,95	1		12	16	20		0,8	0,29	16,0	4,00
Pf-$apitRNA^{Ser}$												
**Acceptor stem**												
G73	1,3	1,1	0,73		16	6,9	6,5		1,0	0,29	9,8	5,37
C73	0,32	0,56	0,66		4,4	3,6	4,4		0,5	0,17	4,1	0,46
U73	0,56	0,71	1,2		3	3,1	6		0,8	0,33	4,0	1,70
G1-C72	1	0,3	2,2		13	10,8	7		1,2	0,96	10,3	3,04
C1-G72	0,35	0,71	0,22		5	6,5	2		0,4	0,25	4,5	2,29
C2-G71/U3-A70	0,6	0,3	0,3		3,6	3	2,4		0,4	0,17	3,0	0,60
**Anticodon triplet**												
C34	10,8	2,45	6	5,5	5,5	2,2	3	2,1	6,2	3,45	3,2	1,59
G35	0,5	0,9	0,55		9	20,8	10		0,7	0,22	13,3	6,54
C35	1,9	1,85	1		3,7	6	2,8		1,6	0,51	4,2	1,65
A35	1,35	2,5	1,22		5,1	3,6	4,7		1,7	0,70	4,5	0,78
C36	0,4	0,9	0,4		2,7	4,3	3,5		0,6	0,29	3,5	0,80
U36	0,8	1,4	0,8		7,3	7,8	5		1,0	0,35	6,7	1,49
G36	1,7	1,5	1		11	11	7,1		1,6	0,36	9,7	2,25
Ser ($C_{35}U_{36}$)	1,3	0,9	1,6		1,73	1,1	1,1		1,3	0,35	1,3	0,36
**Variable region (Vr)**												
ΔVr	1,8	0,85	2,85		5	3,5	5,1		1,8	1,00	4,5	0,90
SerVr	1,4	0,6	0,42	0,8	7	3	4,4	8	0,8	0,43	5,6	2,30
**D-loop (D-l)**												
U20B	0,43	1	1,3		11	8,5	5		0,9	0,44	8,2	3,01
SerVr+D-l	1	0,7	1,25		10	7	5,3		1,0	0,28	7,4	2,38

C

	kcat/KM(1)	kcat/KM(2)	kcat/KM(3)	kcat/KM(4)	Means (kcat/Km)	Loss	p-value
E. coli tRNA^{Tyr}	8,4	7,9	23,0	17,1	14,1	1,5	0,197
Pf-apitRNA^{Tyr}	25,0	16,8	20,0		20,6	1,0	
Pf-apitRNA^{Ser}							
**Acceptor stem**							
G73	12,3	6,3	8,9		9,2	2,2	0,021
C73	13,8	6,4	6,7		8,9	2,3	0,026
U73	5,4	4,4	5,0		4,9	4,2	0,021
G1-C72	13,0	36,0	3,2		17,4	1,2	0,793
C1-G72	14,3	9,2	9,1		10,8	1,9	0,033
C2-G71/U3-A70	6,0	10,0	8,0		8,0	2,6	0,019
**Anticodon triplet**							
C34	0,5	0,9	0,5	0,4	0,6	36,0	0,014
G35	18,0	23,1	18,2		19,8	1,0	0,786
C35	1,9	3,2	2,8		2,7	7,7	0,015
A35	3,8	1,4	3,9		3,0	6,8	0,011
C36	6,8	4,8	8,8		6,8	3,0	0,015
U36	9,1	5,6	6,3		7,0	3,0	0,016
G36	6,5	7,3	7,1		7,0	3,0	0,028
Ser ($C_{35}U_{36}$)	1,3	1,2	0,7		1,1	19,1	0,014
**Variable region (Vr)**							
ΔVr	2,8	4,1	1,8		2,9	7,1	0,012
SerVr	5	5	10,5	10	7,6	2,7	0,013
**D-loop (D-l)**							
U20B	25,6	8,5	3,8		12,6	1,6	0,353
SerVr+D-l	10	10	4,2		8,1	2,1	0,047
